# Directed synthesis of a hybrid improper magnetoelectric multiferroic material

**DOI:** 10.1038/s41467-021-25098-1

**Published:** 2021-08-16

**Authors:** Tong Zhu, Fabio Orlandi, Pascal Manuel, Alexandra S. Gibbs, Weiguo Zhang, P. Shiv. Halasyamani, Michael A. Hayward

**Affiliations:** 1 Department of Chemistry, University of Oxford, Inorganic Chemistry Laboratory, Oxford, UK; 2 ISIS Facility, Rutherford Appleton Laboratory, Chilton, UK; 3 Department of Chemistry, University of Houston, Houston, TX USA

**Keywords:** Electronic materials, Electronic properties and materials

## Abstract

Preparing materials which simultaneously exhibit spontaneous magnetic and electrical polarisations is challenging as the electronic features which are typically used to stabilise each of these two polarisations in materials are contradictory. Here we show that by performing low-temperature cation-exchange reactions on a hybrid improper ferroelectric material, Li_2_SrTa_2_O_7_, which adopts a polar structure due to a cooperative tilting of its constituent TaO_6_ octahedra rather than an electronically driven atom displacement, a paramagnetic polar phase, MnSrTa_2_O_7_, can be prepared. On cooling below 43 K the Mn^2+^ centres in MnSrTa_2_O_7_ adopt a canted antiferromagnetic state, with a small spontaneous magnetic moment. On further cooling to 38 K there is a further transition in which the size of the ferromagnetic moment increases coincident with a decrease in magnitude of the polar distortion, consistent with a coupling between the two polarisations.

## Abstract (web summary)

Fabricating materials with simultaneously spontaneous magnetic and electrical polarisations is challenging due to contradictory electronic features. Here, the authors report a synthesis path toward a perovskite MnSrTa_2_O_7_ by performing low-temperature cation-exchange reactions on Li_2_SrTa_2_O_7_.

## Introduction

Magnetoelectric multiferroic materials^1,2^—those which exhibit spontaneous and coupled magnetic and electrical polarisations—offer the prospect of preparing a range of devices for the manipulation and storage of digital information, including low-power nonvolatile random-access memories^3,4^. However, the preparation of magnetoelectric multiferroic materials is hampered by the contrasting electronic structures typically required for ferroelectric and ferromagnetic behaviour^5^. Specifically, the noncentrosymmetric, polar crystal structures, which are a prerequisite for ferroelectric materials^6,7^, are typically stabilised by second-order Jahn–Teller distortions^8^ of either d^0^ transition metals^9–11^ or *n*s^2^ post-transition metals^12–15^, and these closed-shell electronic configurations are not consistent with magnetism.

The “hybrid improper” (HIP) mechanism offers an alternative method for inducing the noncentrosymmetric polar crystal structures necessary for ferroelectric behaviour^16–20^. In this mechanism, two nonpolar structural distortions, typically the low-energy tilts of *B*O_6_ octahedral units in *A*_3_*B*_2_O_7_ and *A*′*AB*_2_O_7_ layered perovskite phases, combine to break the inversion symmetry of the host framework and couple to a third polar-distortion mode, leading to a spontaneous electrical polarisation^21–28^. A key feature of the trilinear coupling between the three distortion modes is that the polar-distortion mode is not the primary order parameter of the ferroelectric-phase transition of this class of material and is unstable in the absence of the two nonpolar distortions, hence the improper label.

As the HIP mechanism does not rely on the presence of d^0^ or *n*s^2^ diamagnetic ions, it should be easier, in principle, to combine magnetic behaviour with this class of ferroelectric. Indeed, one of the first materials predicted to exhibit HIP ferroelectric behaviour, Ca_3_Mn_2_O_7_^16^, adopts a canted antiferromagnetic state (weak ferromagnet) below 115 K^29^, in which magnetoelectric coupling has been observed^24,30^, in line with theory predictions^16^.

However, further investigation of the Ruddlesden–Popper and Dion–Jacobson phases that exhibit HIP ferroelectric behaviour reveals that large *B*-site cations are needed to stabilise the highly distorted perovskite frameworks required for the HIP mechanism to function^21–27^. When combined with the high *B*-site charges needed in these frameworks, this size requirement rules out the inclusion of the vast majority of paramagnetic transition-metal cations, and to date, the only candidate magnetoelectric multiferroic materials based on the HIP mechanism are Ca_3_Mn_2_O_7_^16,24,30^ and [Ca_0.69_Sr_0.46_Tb_1.85_Fe_2_O_7_]_0.85_[Ca_3_Ti_2_O_7_]_0.15_^31^, the latter of which uses the introduction of some large diamagnetic Ti^4+^ ions onto the perovskite *B* site, via alloying the magnetic iron phase with diamagnetic Ca_3_Ti_2_O_7_, to help stabilise the required distorted perovskite framework.

Recently we have been using the facile cation-exchange chemistry of the *A*′*AB*_2_O_7_ Dion-Jacobson phases to tune the structural distortions of this family of materials to induce HIP ferroelectric behaviour^20,27,32–35^. Here we describe how this cation-exchange chemistry can be used to substitute paramagnetic cations onto the *A*-sites of HIP ferroelectric Ruddlesden–Popper oxides to yield an additional class of magnetoelectric multiferroic materials.

## Results and discussion

**Fig. 1:**
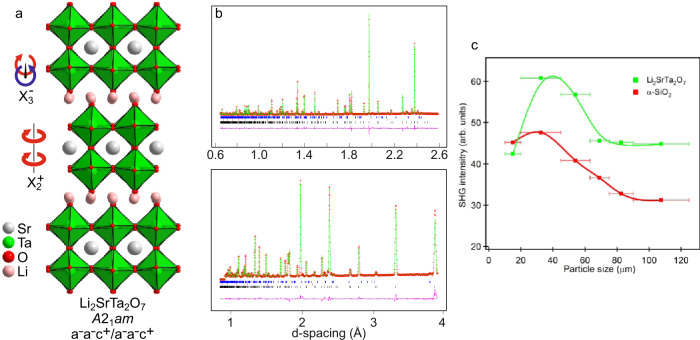
The polar oxide Li_2_SrTa_2_O_7._ **a** The polar crystal structure of Li_2_SrTa_2_O_7_ viewed down the [110] direction. **b** Observed (red crosses), calculated (green line), and difference (purple line) plots from the structural refinement of Li_2_SrTa_2_O_7_ against NPD data. Black, red, and blue tick marks indicate peak positions for the majority phase, the vanadium sample holder and a small quantity of Li_3_TaO_4_, respectively. **c** Particle-size dependent SHG data from Li_2_SrTa_2_O_7_ compared with an α-SiO_2_ standard.

Li_2_SrTa_2_O_7_ was prepared by a ceramic synthesis route from a combination of Li_2_CO_3_, SrCO_3_, and Ta_2_O_5_. Neutron powder diffraction (NPD) data indicate that Li_2_SrTa_2_O_7_ adopts a polar a^–^a^–^c^+^/a^–^a^–^c^+^ distorted, *n* = 2 Ruddlesden–Popper structure, described in space group *A*2_1_*am*, in which the lithium cations reside in pseudo tetrahedral coordination sites between the SrTa_2_O_7_ perovskite double sheets (Fig. 1a). Particle-size-dependent optical SHG data collected at room temperature^36^ (Fig. 1c) are consistent with this noncentrosymmetric structure, in contrast to previous reports^37^. Analysis of the structure of Li_2_SrTa_2_O_7_ reveals that it can be related to the undistorted aristotype Ruddlesden–Popper structure (space group *I*4/*mmm*) by the application of the X_3_^–^ and X_2_^+^ distortion modes that tilt the TaO_6_ octahedra (Fig. 1a), and the Γ_5_^–^ polar-distortion mode, consistent with trilinearly coupled hybrid improper ferroelectric behaviour^16^.

**Fig. 2:**
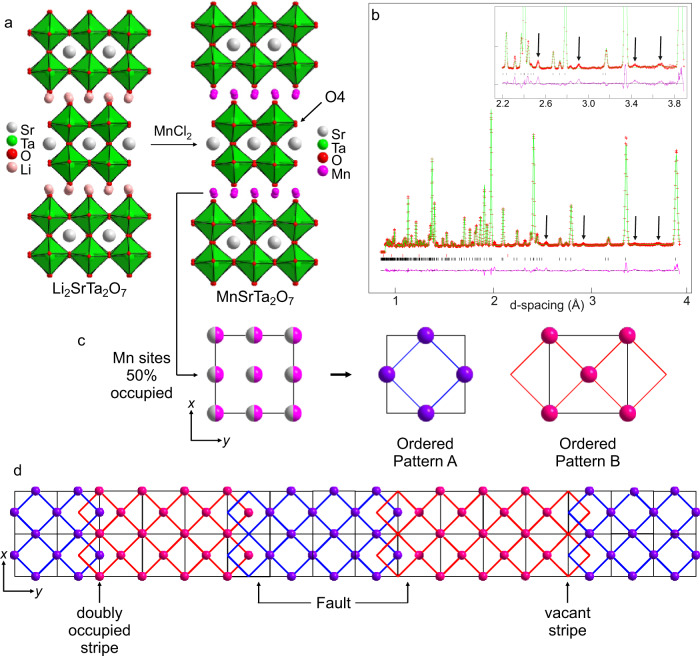
Polar, incommensurate structure of MnSrTa_2_O_7._ **a** Reaction with MnCl_2_ converts Li_2_SrTa_2_O_7_ into MnSrTa_2_O_7_ via topochemical Mn-for-Li cation exchange. **b** Observed (red crosses), calculated (green line), and difference (purple line) plots from refinement of an *A*2_1_*am* symmetry structural model against NPD data collected from MnSrTa_2_O_7_ at 300 K. A series of peaks (marked by arrows) are not fit by this model and can only be indexed with an incommensurate unit cell. **c** Half occupancy of the pseudo tetrahedral coordination sites by Mn^2+^ allows two different local chequerboard orderings. **d** An approximate representation of the incommensurate intergrowth of the two Mn-ordering patterns, which propagates along the crystallographic *y* axis. Locally ordered regions are joined by doubly occupied/vacant stripes or “faults” which run parallel to the *x* axis.

MnSrTa_2_O_7_ was prepared by reacting Li_2_SrTa_2_O_7_ with MnCl_2_ at 375 °C under an inert atmosphere to prevent the conversion of MnCl_2_ to MnO or Mn_3_O_4_. NPD data (HRPD instrument) collected from the cation-exchanged material at room temperature were initially indexed using a modified unit cell based on that of Li_2_SrTa_2_O_7_ and fit using a structural model based on Li_2_SrTa_2_O_7_ (space group *A*2_1_*am*), but with the Li^+^ cations replaced by a 50% occupancy of Mn^2+^ ions, as shown in Fig. 2a. Refinement of this model gave a good fit to the NPD data, with a refined Mn occupancy of 0.496(6), consistent with the stated chemical formula, and the observed SHG activity (0.26 times α-SiO_2_, Supplementary Fig. 2) of the material is consistent with the noncentrosymmetric structure, described in detail in the Supplementary Information.

Close inspection of the fit of this commensurate model to the NPD data revealed a series of weak diffraction peaks that could not be indexed by this model and that could not be assigned to impurity phases (Fig. 2b). However, these additional diffraction features could be indexed using an incommensurate unit cell in superspace group *A*2_1_*am*(0β0)000 with a propagation vector **q** = (0, 0.86, 0) compared with the previously refined commensurate *A*2_1_*am* symmetry unit cell—a situation that is clearer in a further NPD data set collected at 200 K using the WISH diffractometer.

A series of test structural models were constructed and refined against the data to establish which components of the MnSrTa_2_O_7_ framework led to the structural modulation. As shown in Fig. 2c, the Mn^2+^ cations can adopt a chequerboard-ordered arrangement within the tetrahedral coordination sites, analogous to the arrangement of Na^+^ cations in NaNdNb_2_O_7_^27^, resulting in an array of apex-linked MnO_4_ units, which maximises the Mn–Mn separation. However, there are two distinct ways of ordering the Mn cations (A and B), which are simply related by switching the occupied and unoccupied tetrahedral sites.

It was found that the best fit to the NPD data was achieved using a model with a Crenel modulation of the Mn^2+^ occupancy, which corresponds to an incommensurate intergrowth of these two ordering patterns, as shown approximately in Fig. 2d, and described in detail in the Supplementary Information^38,39^. In addition, there is a coupled modulation of the “equatorial” O4 oxide ions that are adjacent to the tetrahedral coordination sites (Fig. 2a), and which move in response to the presence or absence of an Mn^2+^ cation in the neighbouring coordination site. The resulting modulated structure is noncentrosymmetric and polar, consistent with the observed room-temperature SHG activity of the phase, with the electrical polarisation aligned parallel to the crystallographic *x* axis.

The incommensurate modulated ordering of the Mn^2+^ cations over the two pseudotetrahedral coordination sites can be rationalised by noting that the commensurate, chequerboard ordering of the Mn cations, which maximises the Mn–Mn separation, is symmetry-incompatible with the polar a^–^a^–^c^+^/a^–^a^–^c^+^ tilting of the TaO_6_ octahedra. This suggests that the observed modulated ordering pattern of the Mn cations is the optimum compromise between these two structural components^32^.

**Fig. 3:**
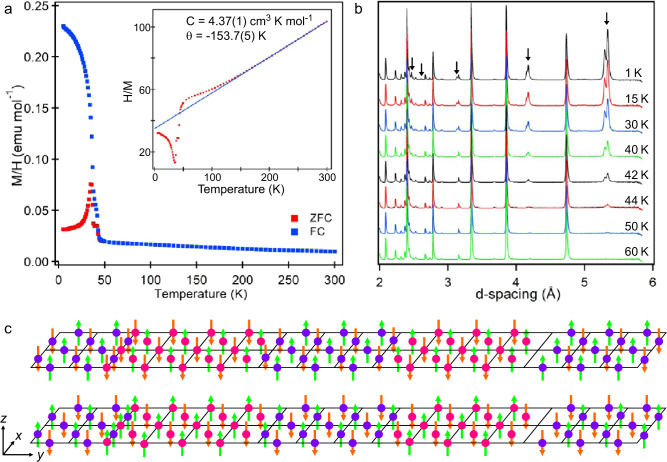
Magnetic behaviour of MnSrTa_2_O_7._ **a** Zero-field-cooled and field-cooled magnetisation data collected from MnSrTa_2_O_7_, the inset shows the fit to the Curie–Weiss law in the range 150 < *T*/K < 300. **b** NPD data collected at indicated temperatures from MnSrTa_2_O_7_. Arrows mark magnetic scattering peaks. **c** The refined magnetic structure of MnSrTa_2_O_7_. Pink and purple spheres represent Mn cations using the colour scheme from Fig. 2, green and orange arrows indicate the direction of the ordered magnetic moments.

Zero-field-cooled (ZFC) and field-cooled (FC) magnetisation data collected from MnSrTa_2_O_7_ (Fig. 3a) can be fit by he Curie–Weiss law in the range 150 < *T*/K < 300 to yield a Curie constant of C = 4.37(1) cm^3^ K mol^−1^ consistent with the value expected for *S* = 5/2, Mn^2+^ cations. On cooling below 43 K, the ZFC and FC data diverge sharply, with field-dependent data exhibiting weak hysteresis at 5 K.

NPD data collected at 1.5 K (Fig. 3b) show a series of strong magnetic Bragg peaks that can be indexed by a single magnetic propagation vector, **k** = (0, 0, 0), of the incommensurate crystallographic unit cell. The best fit to the magnetic diffraction data was achieved using a model described in magnetic space group *A*2_1_*a’m’*(0β0)000, shown in Fig. 3c, in which the Mn moments (4.06(1) µ_B_ at 1.5 K) are aligned parallel to the *z*-axis and ordered in an approximate G-type antiferromagnetic structure that is then perturbed by the modulated Mn cation order. During the refinement of the magnetic model, the crystallographic model was also simultaneously refined, and the structural modulation vector **q** = (0, 0.864, 0) was observed to be unchanged.

Magnetisation data indicate that there is a small ferromagnetic component (canting) to the magnetic structure (0.04 μB per Mn at 5 K). The magnetic space group (*A*2_1_*a’m’*(0β0)000) allows a ferromagnetic component parallel to the *x* axis, consistent with this observation. Furthermore, coupling between the antiferromagnetic order (which transforms as the mX_3_^+^ irreducible representation) and the X_2_^+^ structural distortion stabilises a weak ferromagnetic moment (which transforms as the mΓ_5_^+^ irreducible representation) via a trilinear invariant, as described in the Supplementary information; thus, the magnetic structure of MnSrTa_2_O_7_ is required to have a weak ferromagnetic component by symmetry. However, the small size of this ferromagnetic component means that it could not be observed in the NPD data.

**Fig. 4:**
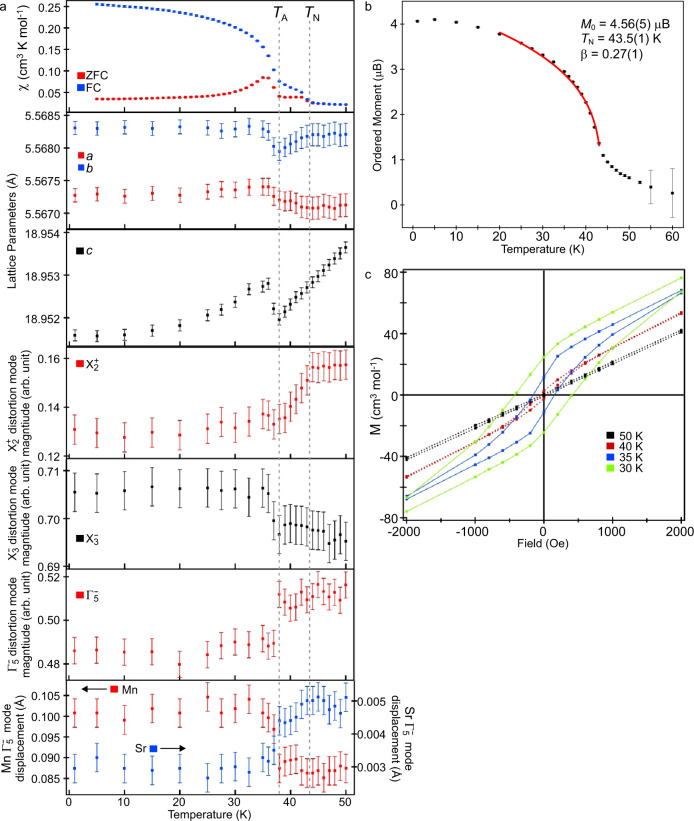
Evidence for magnetoelectric coupling in MnSrTa_2_O_7_. **a** Zero-field-cooled and field-cooled magnetisation data, lattice parameters, X_2_^+^, X_3_^–^, and Γ_5_^–^ distortion-mode magnitude (arbitrary units)^45, 46^ and Mn and Sr Γ_5_^–^ mode displacement of MnSrTa_2_O_7_ in the temperature range 0 < *T*/K < 50. **b** $M={M}_{0}{\left(1-\frac{T}{{T}_{N}}\right)}^{\beta }$ power law fit to ordered Mn moment (obtained by NPD data) as a function of temperature. **c** Expanded region around H = 0 of magnetisation-field data collected from MnSrTa_2_O_7_ in an applied field in the range −50000 < *H*/Oe < 50000.

The temperature dependence of the ordered moment obtained from NPD data can be fitted by a power law, $M={M}_{0}{\left(1-\frac{T}{{T}_{N}}\right)}^{\beta }$, to yield a Néel temperature of *T*_N_ = 43.5(1) K, as shown in Fig. 4b. However, it can be seen from the data in Fig. 3b that magnetic scattering persists above this temperature. Close inspection of the data reveals that above 43 K, the magnetic scattering peaks broaden significantly as they diminish in intensity, and diffuse scattering features appear in the data, as highlighted in the Supplementary Information. This peak broadening/diffuse scattering is attributed to the persistence of strong 2D magnetic interactions in the *xy* plane above *T*_N_. Indeed, the value of the exponential term obtained from the power-law fit (*β* = 0.27(1)) is in excellent agreement with other systems that show strong 2D magnetic interactions, but weak 3D coupling^40^.

As noted above, the ZFC and FC magnetisation data diverge at *T*_N_ = 43 K (Fig. 4a). However, these data show a further feature at 38 K, marked *T*_A_, where the gradient of the ZFC and FC data increases sharply. Field-dependent magnetisation data (Fig. 4c) collected at 40 K, (between *T*_N_ and *T*_A_) are nonlinear and exhibit weak hysteresis, consistent with a canted antiferromagnetic state below *T*_N_. Equivalent data collected at 35 K and 30 K (below *T*_A_) show a significant increase in both remnant magnetisation and coercive field consistent with a sharp increase in the ferromagnetic component (increased canting) of the magnetic state.

Signatures of the event at *T*_A_ are also observed in the crystallographic data, with both the *b* and *c* lattice parameters exhibiting sharp minima at this temperature (Fig. 4a). A more detailed understanding of the structural changes occurring around *T*_N_ and *T*_A_ can be gained by analysing the changes to the magnitudes of the tilting distortion modes, X_3_^–^ and X_2_^+^, and the polar distortion mode, Γ_5_^–^, which relates the *a*^–^*a*^–^*c*^+^/*a*^–^*a*^–^*c*^+^ distorted structure of MnSrTa_2_O_7_ to the corresponding undistorted aristotype structure.

The X_2_^+^ mode, which corresponds to the *a*^0^*a*^0^*c*^+^/*a*^0^*a*^0^*c*^+^ tilting distortion of the TaO_6_ octahedra, is temperature independent from 50 K to *T*_N_ and then exhibits an almost linear decline of around 15% from *T*_N_ to *T*_A_, before becoming essentially temperature independent below *T*_A_, as shown in Fig. 4a. This suggests that while the X_2_^+^ mode responds to the onset of magnetic order at *T*_N_, changes to the magnitude of this mode are not responsible for the minima in the lattice parameters at *T*_A_.

In contrast, the X_3_^–^ mode and the Γ_5_^–^ polar distortion mode are essentially temperature independent between 50 K and *T*_A_, and thus do not appear to respond to the onset of magnetic order at *T*_N_. However, on cooling below *T*_A_, both modes show a step change: an increase of ~1% in X_3_^–^ and a sharp drop of around 5% in the magnitude of the Γ_5_^–^ mode, the latter change indicating a drop in the electrical polarisation of MnSrTa_2_O_7_ at this temperature (Fig. 4a). The drop in the magnitude of the Γ_5_^–^ mode can also be seen in sharp changes to the polar displacements of the Mn and Sr cations, also shown in Fig. 4a. The polar displacement of the Sr cations increases, while that of the Mn cations decreases, because the displacements of the Mn cations in the Γ_5_^–^ distortion mode oppose the establishment of a net polarisation as MnSrTa_2_O_7_ can be thought of as a ferrielectric rather than a ferroelectric.

Taking all these observations together, we observe that at 300 K, MnSrTa_2_O_7_ adopts an incommensurate polar structure (although we note the incommensurate modulation of the structure is not the origin of the inversion symmetry breaking) in a paramagnetic state. On cooling below *T* ~ 100 K, 2-dimensional magnetic correlations build up, before the system undergoes a transition to 3-dimensional magnetic order at *T*_N_ = 43 K. On cooling to *T*_A_ = 38 K, there is a further transition in which the magnitude of the ferromagnetic moment of the phase increases, the X_3_^−^ distortion increases, and the Γ_5_^–^ polar mode decreases.

A symmetry analysis of MnSrTa_2_O_7_ (see Supplementary Information) shows that the mΓ_5_^+^ weak ferromagnetic moment (which arises from the trilinear invariant of the primary antiferromagnetic mX_3_^+^ mode and the X_2_^+^ octahedral tilting) is directly coupled to the Γ_5_^–^ (HIP) polarisation through a quadrilinear invariant involving the mΓ_5_^+^, Γ_5_^–^, mX_3_^+^ and the X_3_^−^ octahedral tilting. Data in Figs. 4a, b show that the magnitudes of the Γ_5_^–^, mX_3_^+^, and X_3_^-^ distortions undergoe a step change at *T*_A_, coincident with a sharp increase in the weak ferromagnetic moment (mΓ_5_^+^) of MnSrTa_2_O_7_, consistent with magnetoelectric coupling.

This quadrilinear coupling also gives an indication that it is possible to reverse both improper ferroic orders, leaving the energy of the system invariant. The actual switching mechanism is more complex and can include different paths, depending on the distortions involved. Indeed, the reversal of the improper ferroelectric polarisation, achievable by the application of an external electric field, requires the reversal of either the X_3_^-^ or the X_2_^+^ octahedral tilting due to the HIP mechanism. If the X_2_^+^ octahedral tilting changes sign, this will also require the reversal of the antiferromagnetic mX_3_^+^ mode or reversal of the weak ferromagnetic moment (mΓ_5_^+^), due to the trilinear invariant coupling of these distortions to the X_2_^+^ tilting. This gives a clear path for the electrical control of the weak ferromagnetic moment via the X_2_^+^ tilting. The reverse mechanism, the magnetic control of the electrical polarisation, is less likely since the application of a magnetic field to switch the weak ferromagnetic moment will presumably result in the change of sign of the mX_3_^+^ antiferromagnetic mode and not in a change of the X_2_^+^ tilting.

The symmetry analysis and couplings detailed above are analogous to those described by Benedek and Fennie for Ca_3_Mn_2_O_7_^16^. However, the appearance of a weak ferromagnetic moment at *T*_N_ prior to the onset/enhancement of magnetoelectric coupling at *T*_A_ does not appear to have an analogue in the observed behaviour of Ca_3_Mn_2_O_7_. Accurate theoretical investigations and switching experiments will be needed to identify the details of the coupling mechanism.

It should be noted that Mn_3_O_4_ exhibits a magnetic ordering transition (*T*_N_ ~ 41−43 K)^41,42^ at a temperature similar to the magnetic events observed for MnSrTa_2_O_7_. However, as shown above, the only features observed in the magnetisation data collected from MnSrTa_2_O_7_ are observed at *T*_N_ and *T*_A_, which can be correlated to features in the NPD data collected from MnSrTa_2_O_7_ (Fig. 4) and attributed to symmetry-required changes of the weak ferromagnetic moment (mΓ_5_^+^) of MnSrTa_2_O_7_. Thus, we can conclude that even if small quantities of Mn_3_O_4_ are present in the sample of MnSrTa_2_O_7_, any contribution they may make to the magnetisation data does not affect the veracity of the analysis presented above. We emphasise that there is no sign of any binary manganese oxide phases in any of the diffraction data collected from any of the samples prepared.

In conclusion, a directed cation-exchange reaction, which replaces the diamagnetic Li^+^ A-cations of the HIP ferroelectric phase Li_2_SrTa_2_O_7_ with paramagnetic Mn^2+^ cations allows the preparation of a coupled magnetoelectric material, MnSrTa_2_O_7_. Given that this type of cation-exchange chemistry should be broadly applicable, this suggests that a wide variety of M^2+^ transition-metal cations can be substituted into polar Li_2_AB_2_O_7_ host phases, allowing the preparation of a large number of additional, potentially magnetoelectric, materials.

## Methods

### Synthesis

Polycrystalline samples of Li_2_SrTa_2_O_7_ were prepared by combining suitable stoichiometric ratios of SrCO_3_ (99.994%) and Ta_2_O_5_ (99.993%, dried at 900 °C) and a 10% stoichiometric excess of Li_2_CO_3_ (99.998%) to compensate for metal loss due to volatility at high temperature. This mixture was then heated at 600 °C in air for 12 h, reground, and pressed into pellets. The pellets were then heated to 1250 °C at a heating rate of 5 °C/min and kept at 1250 °C for 12 h, followed by cooling at 5 °C/min to room temperature. Samples were then reground, pressed into pellets, and heated at 1250 °C for 3 h, before being quenched to room temperature. Polycrystalline samples of MnSrTa_2_O_7_ were prepared by reacting Li_2_SrTa_2_O_7_ with 5-mole equivalents of anhydrous MnCl_2_ (98%). The mixture was ground together in an agate pestle and mortar in an argon-filled glovebox, loaded in a Pyrex tube, and heated for four days at 375 °C under flowing argon. The mixture was then washed with distilled water to remove the remaining chlorides, and dried for 12 h at 140 °C in air.

### Characterisation

Reaction progress and the final sample purity were assessed using X-ray powder diffraction data collected using a PANalytical X’pert diffractometer incorporating an X’celerator position-sensitive detector (monochromatic Cu K*α*_1_ radiation). High-resolution synchrotron X-ray powder diffraction (SXRD) data were collected using instrument I11 at Diamond Light Source Ltd. using Si-calibrated X-rays with an approximate wavelength of 0.825 Å, from samples sealed in 0.3 mm-diameter borosilicate glass capillaries. Low-temperature X-ray powder diffraction data were collected using Rigaku Smartlab diffractometer fitted with a Ge crystal monochromator (Cu, K_α1_) and an Oxford Cryosystems Phenix cryostat. Neutron powder diffraction (NPD) data were collected on the HRPD and WISH diffractometers at the ISIS neutron source, with the samples contained in indium-sealed vanadium cans. Rietveld profile refinements were performed using JANA2006^43^ and the GSAS suite of programs^44^. Symmetry analysis and distortion-model quantification was performed using the ISODISTORT software^45,46^.

Magnetisation data were collected using a Quantum Design MPMS-XL SQUID magnetometer. Zero-field-cooled (ZFC) and field-cooled (FC) data were collected in an applied field of 100 Oe. Powder second harmonic generation measurements were performed on a Kurtz-NLO system using a Nd:YAG laser with a wavelength of 1064 nm. The SHG signal was recorded and compared with a standard sample of *α*-SiO_2_. A detailed description of the experimental setup and process has been reported elsewhere^36^.

## Supplementary information

Supplementary Information

## Data Availability

NPD data are available at 10.5286/ISIS.E.RB1910148. Other data are available on request to the authors.
