# Supplementary material for: Directed synthesis of a hybrid improper magnetoelectric multiferroic material
Source: Nat Commun. 2021 Aug 16;12:4945. doi: 10.1038/s41467-021-25098-1 (PMC8368162; doi:10.1038/s41467-021-25098-1)
Supplement: Supplementary file 1 — Supplementary Information [file 41467_2021_25098_MOESM1_ESM.pdf]

Directed Synthesis of a Hybrid Improper Magnetoelectric Multiferroic Material

Tong Zhu, Fabio Orlandi, Pascal Manuel, Alexandra S. Gibbs, Weiguo Zhang, P. Shiv. Halasyamani and Michael A. Hayward*

Supplementary Information

1. Structural characterisation of $Li_2SrTa_2O_7$.

Supplementary Figure 1. Observed, calculated and difference plots from the refinement of an *A*2$_1$*am* model against high-resolution NPD data collected from $Li_2SrTa_2O_7$ at 298 K.
Supplementary Table 1. Parameters from the structural refinement of $Li_2SrTa_2O_7$ against neutron powder diffraction data collected at 298 K
Supplementary Table 2. Selected bond lengths and bond valence sums from the refined structure of $Li_2SrTa_2O_7$ at 298 K.

2. Structural characterisation of $MnSrTa_2O_7$ – unmodulated model

Supplementary Figure 2. Powder SHG data collected from $MnSrTa_2O_7$ using a 1064 nm pulse Nd:YAG laser. Plot shows the SHG intensity plotted as a function of time, compared with an α–SiO_2 standard.
Supplementary Figure 3. Observed, calculated and difference plots from the refinement of a commensurate *A*2$_1$*am* model against neutron diffraction data collected from $MnSrTa_2O_7$ at room temperature using the HRPD instrument.
Supplementary Figure 4. A plot of the XRD data collected from $MnSrTa_2O_7$ at room temperature.

3. Structural characterisation of $MnSrTa_2O_7$ – modulated model

Supplementary Figure 5. The refined crystal structure of $MnSrTa_2O_7$ using an unmodulated model in space group *A*2$_1$*am*.
Supplementary Figure 6. A comparison of unmodulated and modulated model fits to a selected region of the NPD data collected from $MnSrTa_2O_7$ at room temperature.
Supplementary Figure 7. Observed, calculated and difference plots from the refinement of an *A*2$_1$*am*(0β0)000 model against high resolution neutron powder diffraction data collected from $MnSrTa_2O_7$ at 298 K.
Supplementary Table 3. Parameters from the structural refinement of an incommensurate modulated model for $MnSrTa_2O_7$ against neutron powder diffraction data collected at 298 K.
Supplementary Table 4. Selected bond lengths from the refined modulated structure of $MnSrTa_2O_7$ at 298 K.
Supplementary Figure 8. Observed, calculated and difference plots from the refinement of an *A*2$_1$*am*(0β0)000 model against neutron powder diffraction data collected from $MnSrTa_2O_7$ at 200 K.
Supplementary Table 5. Parameters from the structural refinement of an incommensurate, modulated model for $MnSrTa_2O_7$ against neutron powder diffraction data collected at 200 K.

4. Low-temperature magnetic and crystallographic characterisation of $MnSrTa_2O_7$

Supplementary Figure 9. Observed, calculated and difference plots from the refinement of an *A*2$_1$*a'm'*(0β0)000 model against neutron powder diffraction data collected from $MnSrTa_2O_7$ at 1.5 K.
Supplementary Table 6. Parameters from the structural and magnetic refinement of $MnSrTa_2O_7$ against neutron powder diffraction data collected at 1.5 K.
Supplementary Figure 10. NPD data collected from $MnSrTa_2O_7$ highlighting the magnetic diffuse scattering observed above T_N, consistent with the persistence of 2D magnetic correlations above this temperature.
Supplementary Figure 11. Observed calculated and difference plots from a fit to X-ray powder diffraction data collected from $MnSrTa_2O_7$ at 12 K, using a commensurate model (space group *A*2$_1$*am*).
Supplementary Figure 12. A plot of lattice parameters as a function of temperature extracted from fits to X-ray powder diffraction data collected from $MnSrTa_2O_7$.

5. Symmetry analysis and coupling invariants

Supplementary Figure 13. The symmetry lowering distortions of $MnSrTa_2O_7$ and their couplings.

1. Structural characterisation of $Li_2SrTa_2O_7$.

Neutron powder diffraction data collected from $Li_2SrTa_2O_7$ at room temperature using the HRPD instrument (ISIS neutron source) could be indexed using an orthorhombic unit cell (a = 5.5784(1) Å, b = 5.5842(1) Å, c = 18.1845(1) Å) with extinction conditions consistent with A-centring. Considering the observed SHG activity of $Li_2SrTa_2O_7$ (Figure 1c, main text) the centrosymmetric structure previously reported for $Li_2SrTa_2O_7$, with an $a^-a^-c^0/a^-a^-c^0$ tilting distortion, described in space group *Amam* cannot be correct.[1] Instead we utilized a non-centrosymmetric structural model with an $a^-a^-c^+/a^-a^-c^+$ tilting distortion, described in space group $A2_1am$. Close inspection of the diffraction data revealed weak diffraction reflections from the vanadium sample holder and a Li_3TaO_4 secondary phase (0.06 wt%) so these were added to the model. The refinement proceeded smoothly to give a good fit. Observed calculated and difference plots from the refinement are shown in Supplementary Figure 1, with a complete description of the structure given in Supplementary Table 1 and selected bond lengths and bond valence sums in Supplementary Table 2.

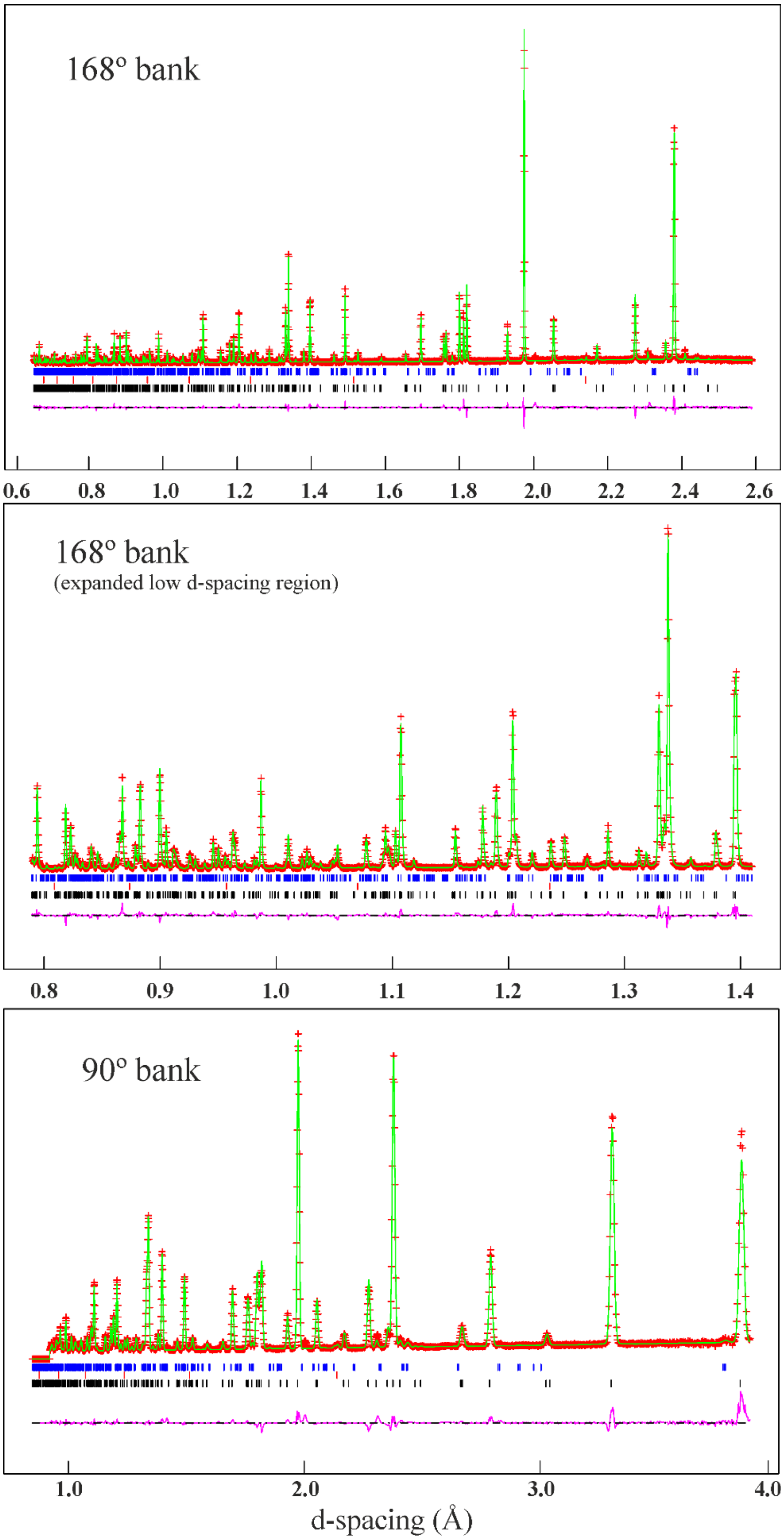

Supplementary Figure 1. Observed, calculated and difference plots from the refinement of an *A*2$_1$*am* model against high-resolution NPD data collected from $Li_2SrTa_2O_7$ at 298 K. Tick marks indicate peak positions of $Li_2SrTa_2O_7$ (black) vanadium (red) and Li_3TaO_4 (blue) respectively.

Atom	Site symmetry	*x*	*y*	*z*	U_{iso} (Å^2)
Li(1)	8*b*	0.4909(27)	0.0106(38)	0.2442(3)	0.0153(14)
Sr(1)	4*a*	0.2275(15)	0.2444(5)	0	0.0071(3)
Ta(1)	8*b*	0.2194(9)	0.7471(4)	0.1149(1)	0.0028(2)
O(1)	4*a*	0.2314(17)	0.7791(5)	0	0.0099(6)
O(2)	8*b*	0.2213(11)	0.7297(4)	0.2174(1)	0.0067(3)
O(3)	8*b*	0.9788(15)	0.4971(14)	0.0968(1)	0.0049(4)
O(4)	8*b*	0.9806(17)	0.0054(12)	0.1121(1)	0.0075(4)
$Li_2SrTa_2O_7$ – space group $A2_1am$ (#36)<br>a = 5.5784(1) Å, b = 5.5842(1) Å, c = 18.1845(1) Å, volume = 566.46(1) Å^3<br>Formula weight: 575.39 g mol^{-1}, Z = 4<br>Radiation source: Neutron Time of flight<br>Temperature: 298 K<br>χ^2 = 10.08, wRp = 5.71%, Rp = 5.35%					

Supplementary Table 1. Parameters from the structural refinement of $Li_2SrTa_2O_7$ against neutron powder diffraction data collected at 298 K

Cation	Anion	length (Å)	BVS
Li(1)	O(2) × 1	2.060(18)	Li +0.81
	O(2) × 1	2.227(19)	
	O(2) × 1	1.984(19)	
	O(2) × 1	1.998(19)	
Sr(1)	O(1)	2.770(13)	Sr +2.21
	O(1)	2.986(5)	
	O(1)	2.599(5)	
	O(1)	2.814(13)	
	O(3) × 2	2.648(8)	
	O(3) × 2	2.673(8)	
	O(4) × 2	2.799(8)	
	O(4) × 2	2.846(8)	
Ta(1)	O(1)	2.099(1)	Ta +5.11
	O(2)	1.867(1)	
	O(3)	1.964(9)	
	O(3)	2.016(9)	
	O(4)	1.964(9)	
	O(4)	2.009(10)	

Supplementary Table 2. Selected bond lengths and bond valence sums from the refined structure of $Li_2SrTa_2O_7$ at 298 K.

2. Structural characterisation of $MnSrTa_2O_7$ – unmodulated model

Synchrotron X-ray and neutron powder diffraction data collected from $MnSrTa_2O_7$ at room temperature (298 K) can be indexed using an orthorhombic unit cell with a $\sqrt{2} \times \sqrt{2} \times 1$ geometric expansion of the aristotype *I*4/*mmm* unit cell (*a* = 5.5672 Å, *b* = 5.5673 Å, *c* = 18.9925 Å), consistent with a distorted *n* = 2 Ruddlesden-Popper type structure. The extinction conditions are consistent with an A-centred structure.

Powder SHG data were collected from $MnSrTa_2O_7$ contained within a fused silica tube (OD = 4 mm) using a 1064 nm pulse Nd:YAG laser. Data shown in Supplementary Figure 2 indicate $MnSrTa_2O_7$ is SHG active yielding a signal 0.26 times the strength of an α-SiO_2 standard at room temperature. It should be noted that the SHG intensity of the sample is particle size dependent, so the absolute value of the SHG intensity is not very informative. Particle-size dependent SHG measurements were not possible for $MnSrTa_2O_7$ as the multiple grinding cycles in the cation exchange reaction used to prepare $MnSrTa_2O_7$ mean that the variation in particle size in the sample is too narrow (not enough particles larger than 50μm) to make this type of measurement meaningful. However, the data present in Supplementary Figure 2 provide an unambiguous indication the $MnSrTa_2O_7$ is SHG active and thus has a non-centrosymmetric crystal structure.

The observed SHG activity, the unit cell size and the extinction conditions are consistent with a structure based on that of $Li_2SrTa_2O_7$ described in the polar space group $A2_1am$. Therefore, a structural model was constructed for $MnSrTa_2O_7$ based on the distorted *n* = 2 Ruddlesden-Popper structure of $Li_2SrTa_2O_7$ (space group $A2_1am$) with the Li^+ cations replaced by a 50% occupancy of Mn^{2+} cations on the 8*b*, pseudo-tetrahedral coordination sites.

Refinement of this $A2_1am$ symmetry model against the room temperature neutron powder diffraction data (HRPD) proceeded smoothly. All atomic positional and displacement parameters were refined freely during the refinement. The manganese occupancy was refined as a last step. The refinement gave a good fit to the data (wRp = 6.07%, Rp = 5.54%) and yielded a refined manganese occupancy of 0.496(6), confirming a complete cation exchange from $Li_2SrTa_2O_7$ to $MnSrTa_2O_7$.

A close inspection of the diffraction data reveals a series of reflections which are not indexed by this model (Supplementary Figure 3). Peaks marked with dashed-arrows are attributed to impurity phases formed during the synthesis of the $Li_2SrTa_2O_7$ precursor phase. Peaks labelled

with solid arrows cannot be indexed by any impurity phase or a further commensurate cell expansion, indicating a possibility of a modulated structure.

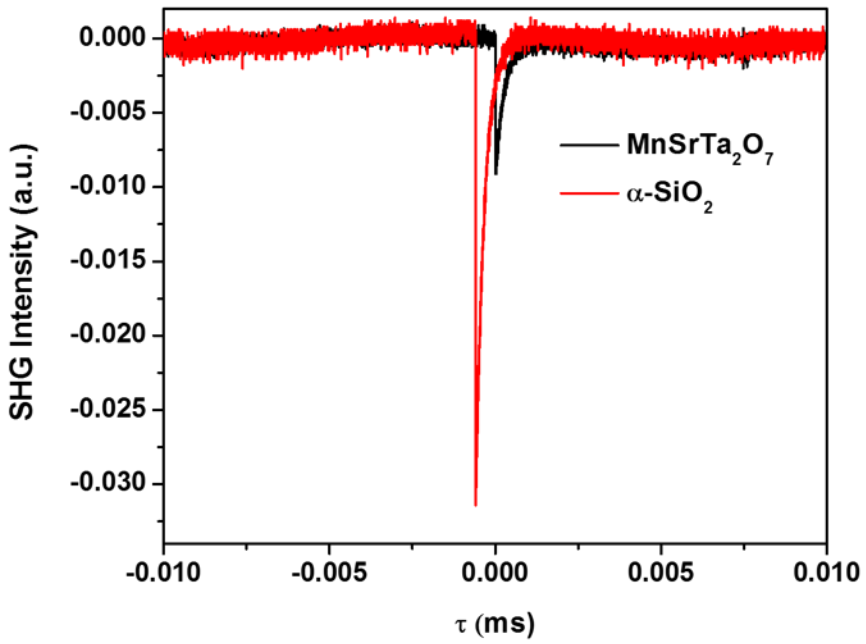

Supplementary Figure 2. Powder SHG data collected from $MnSrTa_2O_7$ using a 1064 nm pulse Nd:YAG laser. Plot shows the SHG intensity plotted as a function of time, compared with an α–SiO_2 standard.

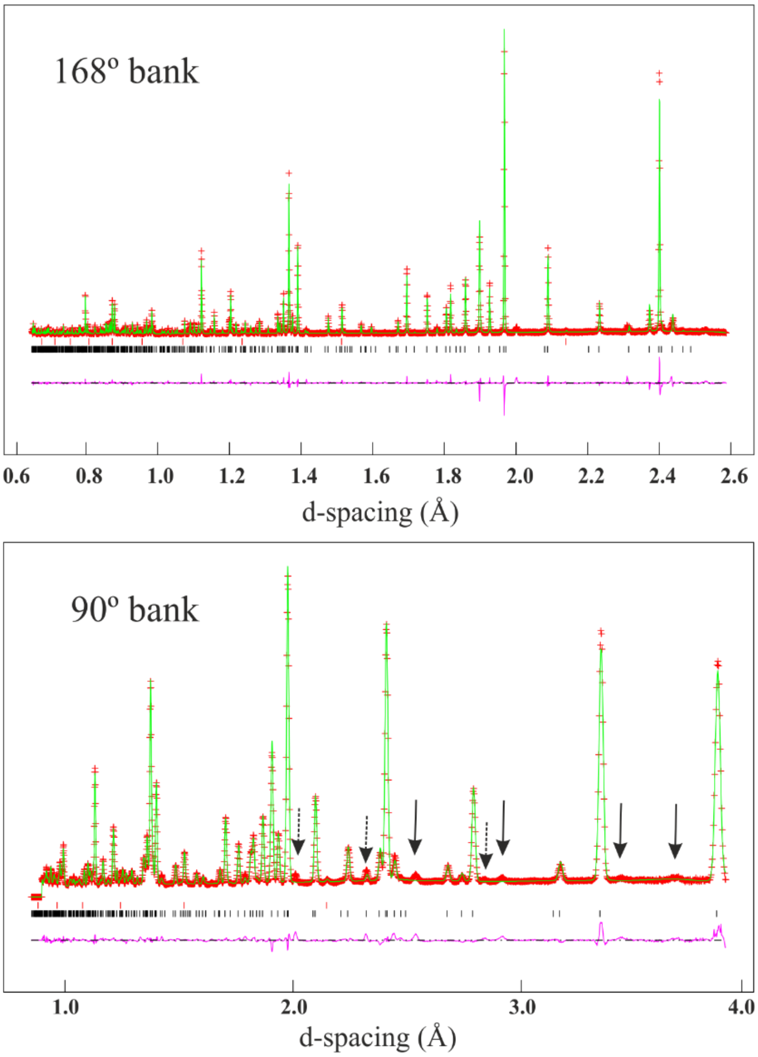

Supplementary Figure 3. Observed, calculated and difference plots from the refinement of a commensurate $A2_1am$ model against neutron diffraction data collected from $MnSrTa_2O_7$ at room temperature using the HRPD instrument. Reflections from the main phase are indexed with black tick-marks contributions from the vanadium sample holder with red tick-marks.

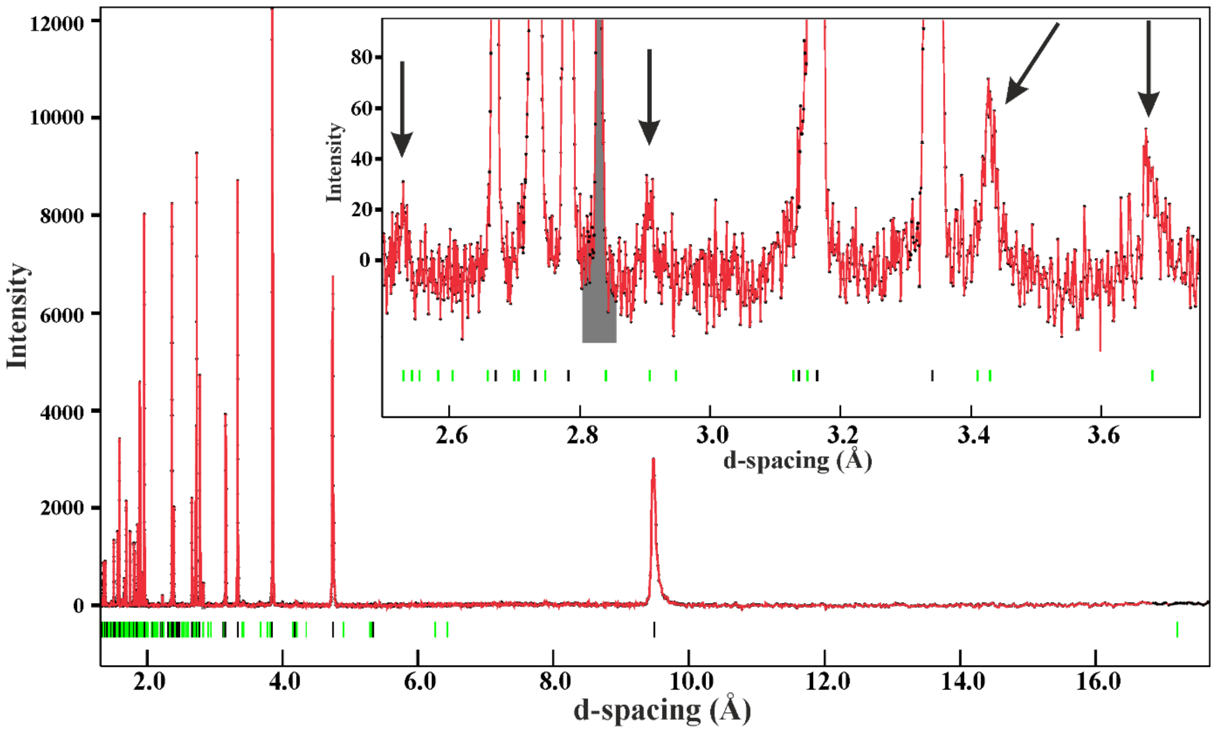

Supplementary Figure 4. A plot of the XRD data collected from $MnSrTa_2O_7$ at room temperature. Black and green tick marks indicate positions of main reflections (commensurate model) and satellite reflections (indexed by modulated cell) respectively. The shaded region indicates the contribution of an impurity phase which forms during the synthesis of the precursor $Li_2SrTa_2O_7$ phase. Arrows indicate the positions of peaks which cannot be indexed by the commensurate unit cell.

3. Structural characterisation of $MnSrTa_2O_7$ – modulated model

In order to better study the possibility that $MnSrTa_2O_7$ has a modulated structure, a further neutron diffraction dataset was collected at 200 K (well above the magnetic transition temperature) using the WISH diffractometer, which covers a larger d-spacing range than the HRPD data. Satellite reflections observed in both the 298 K (HRPD) and 200 K (WISH) data sets, which are not indexed by the unmodulated $A2_1am$ model, can be indexed by a cell in superspace group $A2_1am(0\beta0)000$ with a propagation vector of $\mathbf{q}$ = (0, 0.86, 0) compared to the previously refined commensurate $A2_1am$ unit cell, consistent with an incommensurate modulated crystal structure for $MnSrTa_2O_7$. The $A2_1am(0\beta0)000$ superspace group could be unambiguously determined by observing the systematic absences *hklm* with $k+l=2n$ and *h0lm* with $h=2n$.

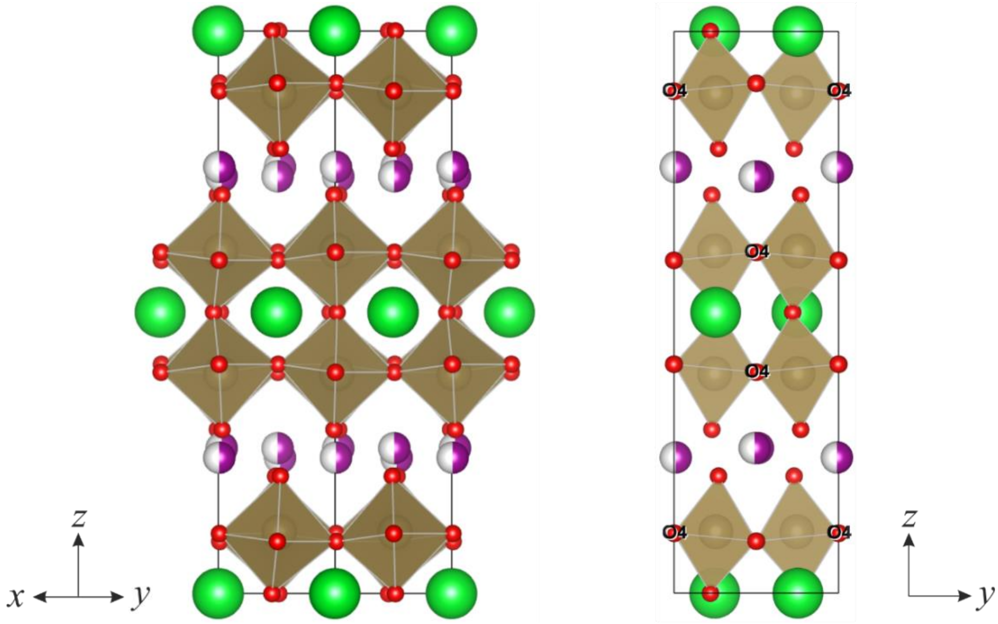

Supplementary Figure 5. The refined crystal structure of $MnSrTa_2O_7$ using an unmodulated model in space group $A2_1am$. The purple/white, green and red spheres represent Mn^{2+}, Sr^{2+} and O^{2-} ions respectively. Ta^{5+} cations reside in the octahedra.

In the average, unmodulated structure of $MnSrTa_2O_7$ (Supplementary Figure 5), there is one Mn (8*b*) site, one Sr (4*a*) site, one Ta (8*b*) site and four O sites. To determine which of these sites was responsible for the modulation of the structure an incommensurate, modulated model in superspace group $A2_1am(0\beta0)000$ was constructed by combining the previously refined $A2_1am$ structure and modulation waves for each atom site (Mn, Sr, Ta, O) individually, with the refinement outcomes carefully compared with the fits using the unmodulated model. These tests revealed that neither occupational nor positional modulation of the Sr and Ta sites

improved the fit to the data. Similarly, refining a positional modulation wave for the Mn cations also gave no improvement of the fit. However, when the occupancy wave of Mn was refined, some satellite peak intensities were fitted (Supplementary Figure 6) which had zero intensity in the unmodulated model. It should be noted that the Mn occupancy modulation wave can be described in two ways: a smooth continuous change of Mn occupancy along *y* direction (the propagation direction) where the Mn occupancy can be any value between zero and unity or a discontinuous variation (Crenel function) where the Mn occupancy can only adopt a value of 1 for $x_4^0 \pm \Delta/2$ or 0 elsewhere, where Δ is the width of the crenel function (which corresponds to the site occupancy) centred in x_4^0 with x_4 being the internal (fourth) coordinate.[2,3] Refinements indicated this latter, discontinuous modulation of the Mn occupancy gave the best fit to the data. However, as shown in Supplementary Figure 6, even when this discontinuous Mn-occupancy modulation is added to the structural model there are still some satellite peak intensities which are not properly fitted, which led us to study the possibility of a modulation of the oxide ions.

Refining anisotropic displacement parameters for the oxide ions revealed that the O(4) anion site has a very large U_{33} value (0.035 Å^2), suggesting a modulation of this anion site. A model was constructed in which the O4 anion site was split into two separate sites, O(4)' and O(4)'' with the occupancies of these two sites described by a discontinuous modulation wave which was constrained so that for each pair of adjacent O4'/O4'' sites, one was occupied and one was unoccupied at all positions in the crystal. The positions of the O4' and O4'' sites refined to locations which differ in their *z* coordinates while both the *x* and *y* coordinates converge to be the same within error, thus during the final refinement the *x* and *y* coordinates were constrained to be the same for O4' and O4'' sites (Supplementary Table 3). As shown in Supplementary Figure 6, adding the O(4) modulation to the model gave a significant improvement of the fit to the data. No obvious improvement was observed when refining a modulation of other oxygen sites (O(1), O(2) and O(3)).

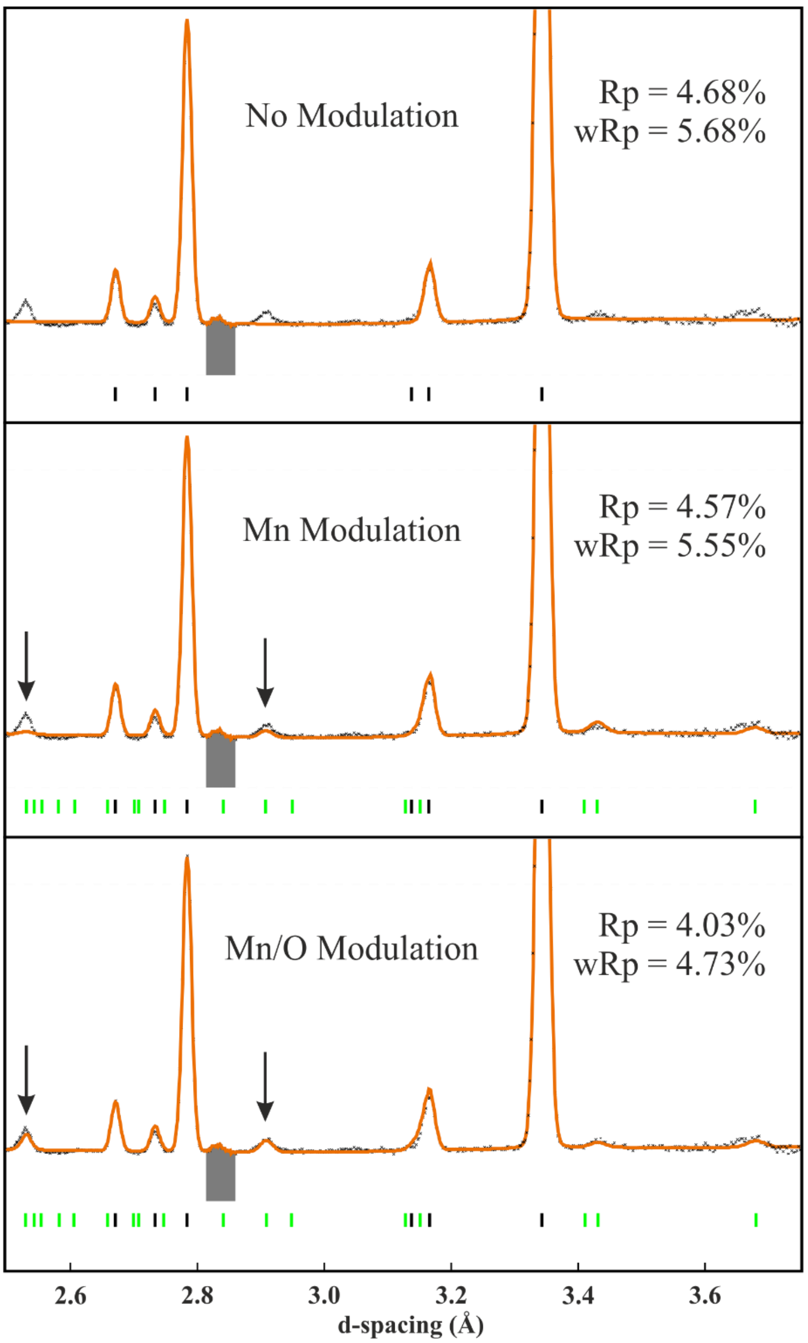

Supplementary Figure 6. A comparison of fits to a selected region of the NPD data collected from $MnSrTa_2O_7$ at room temperature. Top, middle and bottom plots correspond to the refinements using an unmodulated model, an incommensurate modulated model with only Mn modulation and an incommensurate modulated model with both Mn and O(4) modulation, respectively. Black and green tick marks indicate positions of main reflections and satellite reflections respectively. Black dots and orange curves represent the diffraction data and simulated patterns respectively. Shaded regions remove the contribution of an impurity phase which forms during the synthesis of the precursor $Li_2SrTa_2O_7$ phase. Peaks labelled with arrows highlight the improvement of fits when refining the extra oxygen modulation wave.

Refinement of the modulated model against NPD data collected at 200 K using the WISH diffractometer indicated a strong correlation between the modulation waves of Mn and O(4) sites, as uncorrelated waves predicted strong peaks which are not observed in the diffraction data, which were especially noticeable at high d-spacing regions. Therefore, constraints are added in the refinements. In particular the x_4^0 values for the Mn, O4 and O4' sites were fixed to be 0.25, 0.25 and 0.75 respectively in our final step refinement. It should be noted that x_4^0 (Mn) of 0.25 is a refined value, while the correlation between the modulation waves gives the following constraint: x_4^0 (Mn) = x_4^0 (O4) = x_4^0 (O4') - 0.5.

This correlated, modulated model was then refined against NPD data (HRPD) to establish the room temperature structure of the phase. The observed, calculated and difference plots from the refinement of the $A2_1am$(0b0)000 model against room temperature NPD data are shown in Supplementary Figure 7. Full details of the refined parameters are listed in Supplementary Table 3, with selected bond lengths in Supplementary Table 4. The observed, calculated and difference plots from the refinement of the $A2_1am$(0β0)000 model against 200K NPD data (WISH) are shown in Supplementary Figure 8 and full details of the parameters from the refinements are shown in Supplementary Table 5.

In light of the polar structure determined for $MnSrTa_2O_7$, attempts were made to make a direct measurement of the polarisation of the material. However, the cation exchange reaction used to prepare $MnSrTa_2O_7$ is only capable of producing powder samples, which are unsuitable for polarisation or pyroelectric current measurements. Furthermore, the metastability of $MnSrTa_2O_7$ prevents the preparation of dense sintered pellets of the material, as we observe significant decomposition of $MnSrTa_2O_7$ at the temperatures required to prepare dense pellets suitable for polarization or pyroelectric measurements.

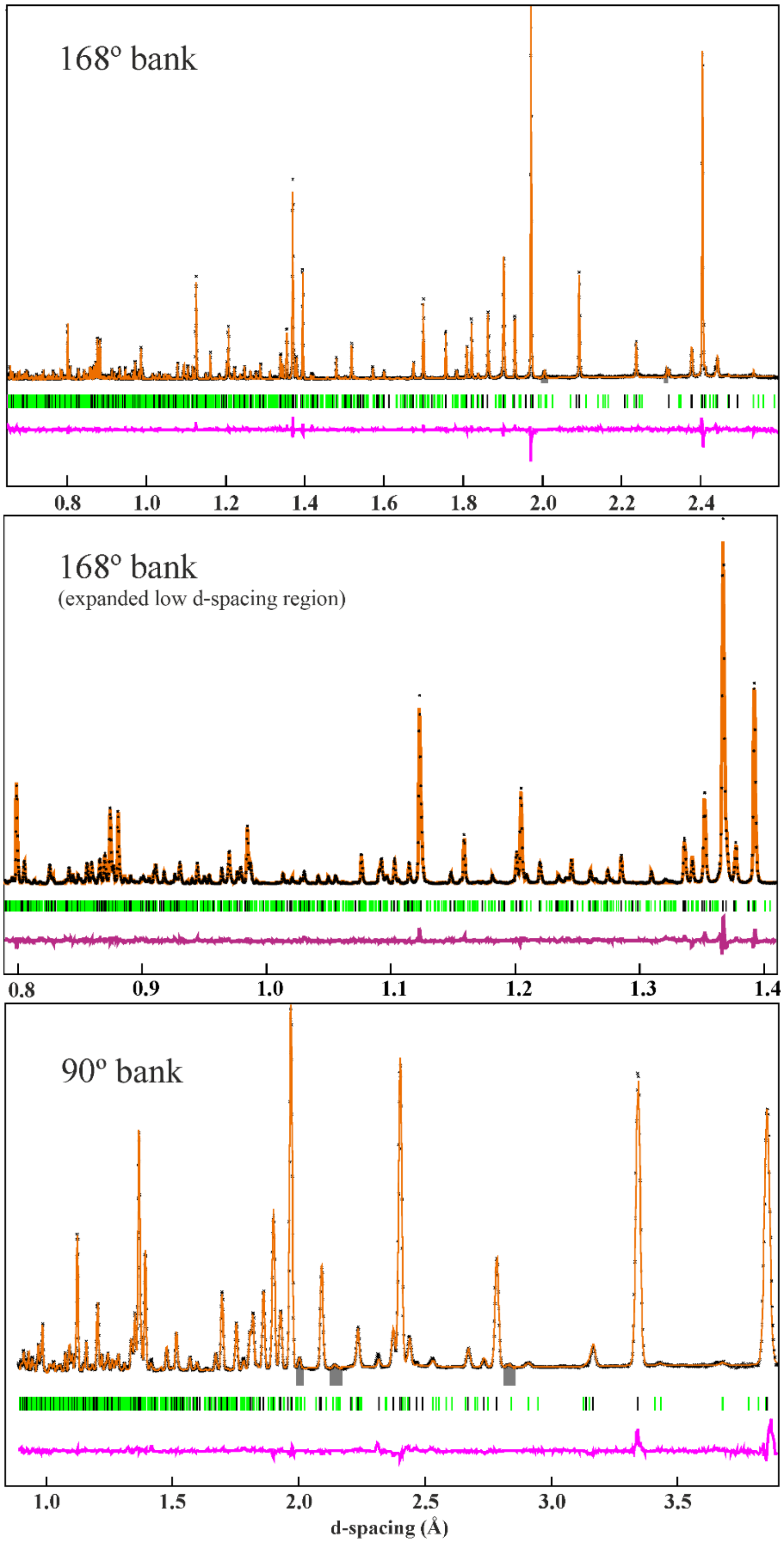

Supplementary Figure 7. Observed, calculated and difference plots from the refinement of an $A2_1am$(0b0)000 model against high-resolution NPD data collected from $MnSrTa_2O_7$ at 298 K (HRPD). Black and green tick marks indicate positions of main reflections and satellite reflections respectively. Shaded regions remove the contribution of an impurity phase which forms during the synthesis of the precursor $Li_2SrTa_2O_7$ phase.

Atom	*x*	*y*	*z*	Fraction	U_{iso} (Å^3)
*Mn(1)	0.4976(25)	0.5029(35)	0.2598(2)	1*	0.0113(8)
Sr(1)	0.2406(12)	0.2439(4)	0	1	0.0070(2)
Ta(1)	0.7416(11)	0.2516(4)	0.1095(1)	1	0.0033(2)
O(1)	0.7595(11)	0.2199(8)	0	1	0.0089(6)
O(2)	0.7377(9)	0.2702(4)	0.2082(1)	1	0.0123(4)
O(3)	0.9881(9)	0.5019(10)	0.0931(1)	1	0.0071(3)
*O(4’)	0.9826(7)	0.9917(9)	0.1146 (1)	1*	0.0040(4)
*O(4’’)	0.9826(7)	0.9917(9)	0.0975(1)	1*	0.0040(4)
$MnSrTa_2O_7$ – superspace group $A2_1am$(0b0)000, q2 = 0.8647(7)<br>Δ (Mn) = Δ (O4) = Δ (O4’) = 0.5<br>x_4^0 (Mn) = x_4^0 (Mn) = 0.25, x_4^0 (O4’) = 0.75<br>*a* = 5.5673(1) Å, *b* = 5.5670(1) Å, *c* = 18.9925(1) Å, volume = 588.632(3) Å^3<br>Formula weight: 615.85 g mol^{-1}<br>Radiation source: Neutron Time of flight<br>Temperature: 298 K<br>GOF = 3.18, wRp = 4.73%, Rp = 4.03%					

Supplementary Table 3. Parameters from the structural refinement of an incommensurate, modulated model for $MnSrTa_2O_7$ against neutron powder diffraction data collected at 298 K (HRPD). Occupancy modulation of Mn and O4 was refined. O4 site needs to split into two in order to describe the real structure.

Cation	Anion	length (Å)	BVS
Mn(1)	O(2) × 1	2.19(6)	Mn +1.79
	O(2) × 1	2.16(6)	
	O(2) × 1	2.09(6)	
	O(2) × 1	2.10(6)	
	O(4') × 1	2.388(5)	
Sr(1)	O(1)	2.681(19)	Sr +2.33
	O(1)	2.99(2)	
	O(1)	2.58(2)	
	O(1)	2.892(19)	
	O(3) × 2	2.677(16)	
	O(3) × 2	2.651(16)	
	O(4') × 2	2.876(13)	
	O(4'') × 2	2.732(14)	
Ta(1)	O(1)	2.090 (3)	Ta +5.11
	O(2)	1.878(3)	
	O(3)	1.98(3)	
	O(3)	1.99(2)	
	O(4')	1.976(19)	
	O(4'')	1.991(19)	

Supplementary Table 4. Selected bond lengths from the refined modulated structure of $MnSrTa_2O_7$ at 298 K.

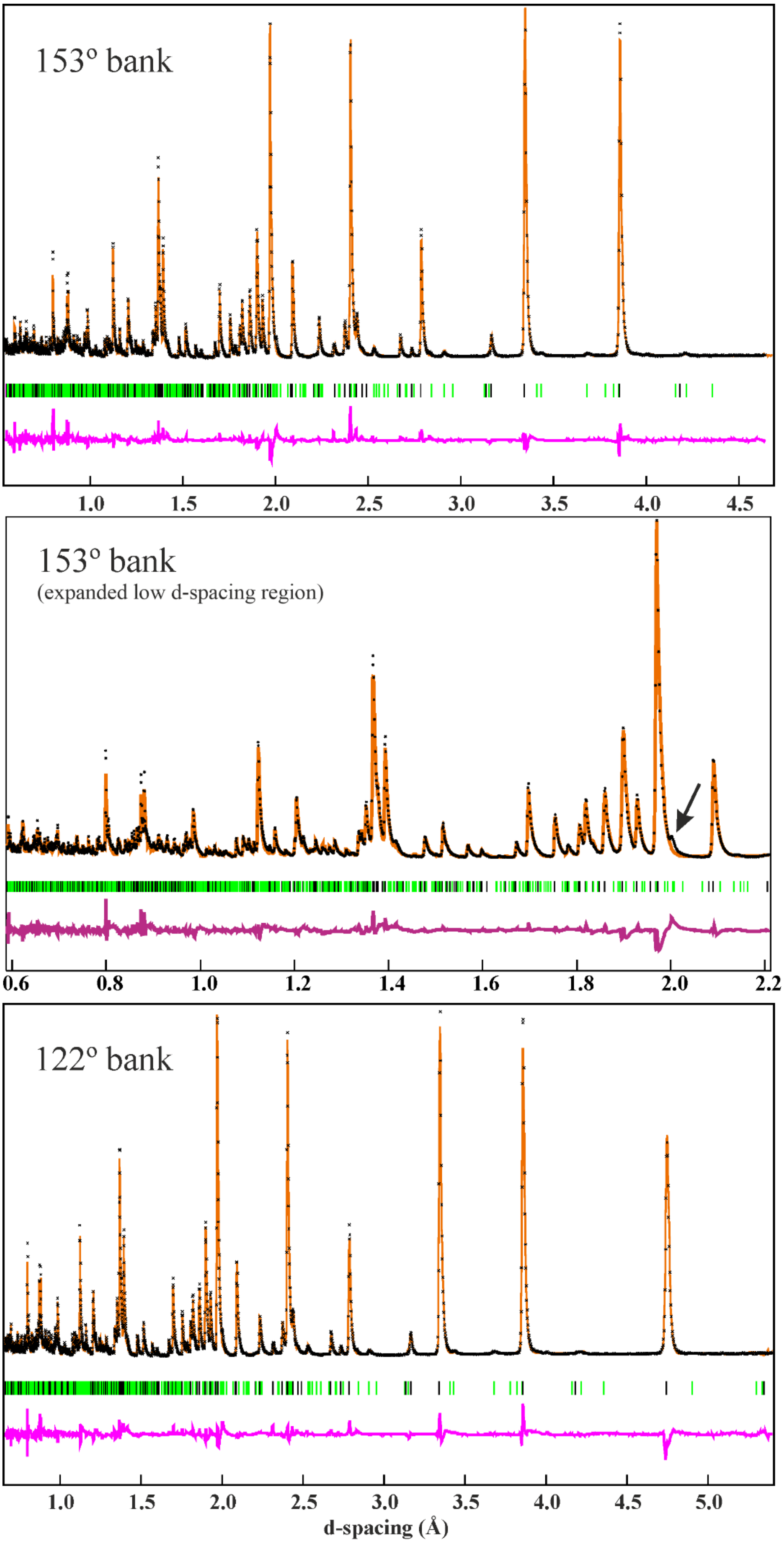
153° bank
1.0
1.5
2.0
2.5
3.0
3.5
4.0
4.5
153° bank
(expanded low d-spacing region)
0.6
0.8
1.0
1.2
1.4
1.6
1.8
2.0
2.2
122° bank
1.0
1.5
2.0
2.5
3.0
3.5
4.0
4.5
5.0
d-spacing (Å)

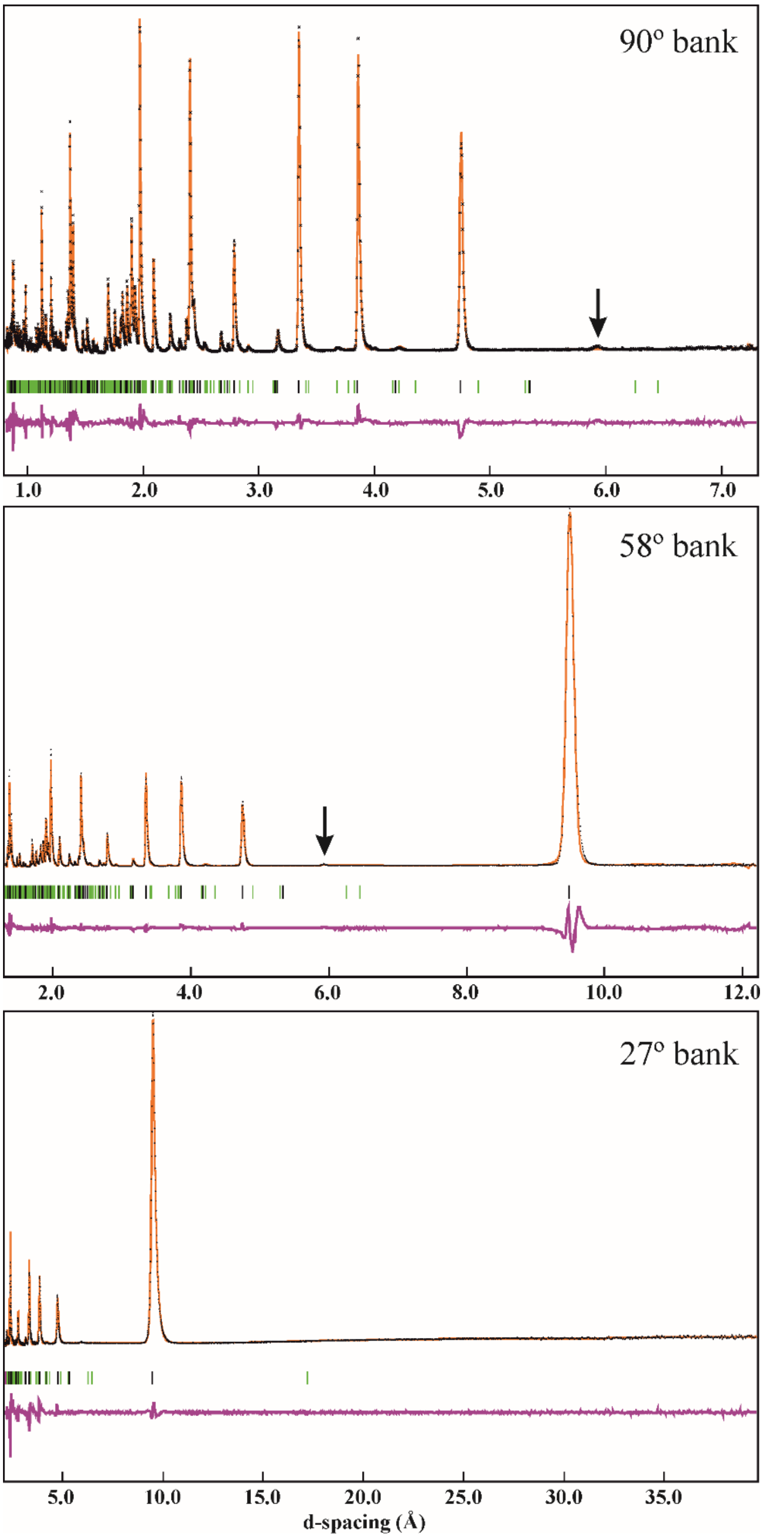

Supplementary Figure 8. Observed, calculated and difference plots from the refinement of an $A2_1am(0\beta0)000$ model against NPD data collected from $MnSrTa_2O_7$ at 200 K (WISH). Black and green tick marks indicate positions of main reflections and satellite reflections respectively. Arrows mark peaks from an impurity 'inherited' from the $Li_2SrTa_2O_7$ starting material.

Atom	*x*	*y*	*z*	Fraction	U_{iso} (Å^3)
*Mn(1)	0.4620(18)	0.4915(30)	0.2608(2)	1*	0.0006(3)
Sr(1)	0.2405(20)	0.2387(8)	0	1	0.0086(5)
Ta(1)	0.7450(18)	0.2502(7)	0.1096(1)	1	0.0076(3)
O(1)	0.7328(22)	0.2262(10)	0	1	0.0177(11)
O(2)	0.7603(16)	0.2705(6)	0.2078(1)	1	0.0095(6)
O(3)	0.9949(16)	0.4975(20)	0.0918(1)	1	0.0082(6)
*O(4')	0.0113(15)	0.0028(13)	0.1180 (1)	1*	0.0025(7)
*O(4'')	0.0113(15)	0.0028(13)	0.0984 (2)	1*	0.0025(7)
$MnSrTa_2O_7$ – superspace group *A*2$_1$*am*(0β0)000, q2 = 0.8635(5)<br>Δ (Mn) = Δ (O4) = Δ (O4') = 0.5<br>x_4^0 (Mn) = x_4^0 (Mn) = 0.25, x_4^0 (O4') = 0.75<br>*a* = 5.5674(1) Å, *b* = 5.5699(1) Å, *c* = 18.9756(1) Å, volume = 588.490(7) Å^3<br>Formula weight: 615.85 g mol^{-1}<br>Radiation source: Neutron Time of flight<br>Temperature: 200 K<br>GOF = 7.44, wRp = 5.20%, Rp = 5.88%					

Supplementary Table 5. Parameters from the structural refinement of an incommensurate, modulated model for $MnSrTa_2O_7$ against neutron powder diffraction data collected at 200 K (WISH). Occupancy modulation wave of Mn and O4 was refined. O4 site needs to split into two in order to describe the real structure.

4. Low-temperature magnetic and crystallographic characterisation of $MnSrTa_2O_7$

Neutron powder diffraction data collected from $MnSrTa_2O_7$ at 1.5 K (WISH) show strong magnetic Bragg peaks, consistent with long-range magnetic order (Figure 3b, main text). These additional peaks can be indexed by a single magnetic propagation vector, **k** = (0, 0, 0), of the incommensurate structure. A series of possible magnetic models was then constructed based on the refined crystal superstructure. Their diffraction patterns were simulated and compared to the diffraction data collected at 1.5 K. The best match to the data was achieved by using the model in magnetic space group $A2_1a'm'(0\beta0)000$.

A magnetic model described in space group $A2_1a'm'(0\beta0)000$ was refined against neutron powder diffraction data collected at 1.5 K. Initially the crystal structure was refined, and was observed to be directly analogous to that obtained from the refinements against data collected at room temperature and 200 K, described above, with an unchanged modulation vector **q** = (0, 0.864, 0). Refinement of the magnetic model revealed that the Mn moments (4.06(1) μ_B) are aligned along *z* with moments along both the *x* and *y* directions converging to zero within error. It should be noted that the magnetization data collected at low temperature (Figure 3a and 4c, main text) indicate a small ferromagnetic component consistent with canted antiferromagnetic order. The symmetry of the magnetic model (magnetic space group $A2_1a'm'(0\beta0)000$) does allow a ferromagnetic component parallel to the *x*-axis. However, the signature of this ferromagnetic component in the neutron diffraction data is too small to be resolved, and hence the refined magnetic structure is a co-linear, uncanted antiferromagnet. The observed, calculated and difference plots from this refinement are shown in Supplementary Figure 9, with a representation of the ordered magnetic model shown in Figure 3c, in the main text, with full details given in Supplementary Table 6.

The crystallographic and magnetic model obtained by refinement against the 1.5 K NPD data was then refined against NPD data collected at temperatures up to 60 K.

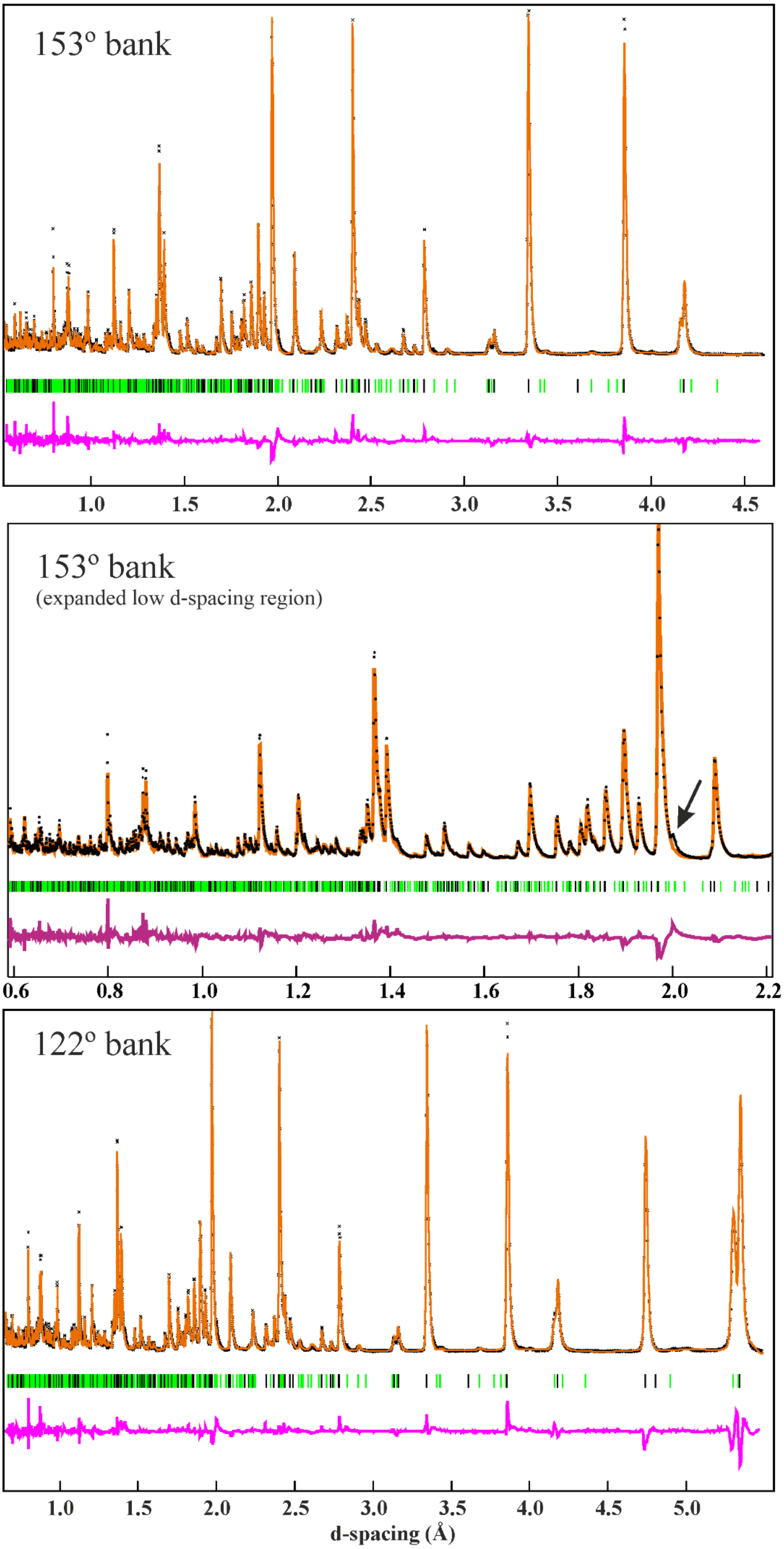

153° bank
1.0 1.5 2.0 2.5 3.0 3.5 4.0 4.5
153° bank
(expanded low d-spacing region)
0.6 0.8 1.0 1.2 1.4 1.6 1.8 2.0 2.2
122° bank
1.0 1.5 2.0 2.5 3.0 3.5 4.0 4.5 5.0
d-spacing (Å)

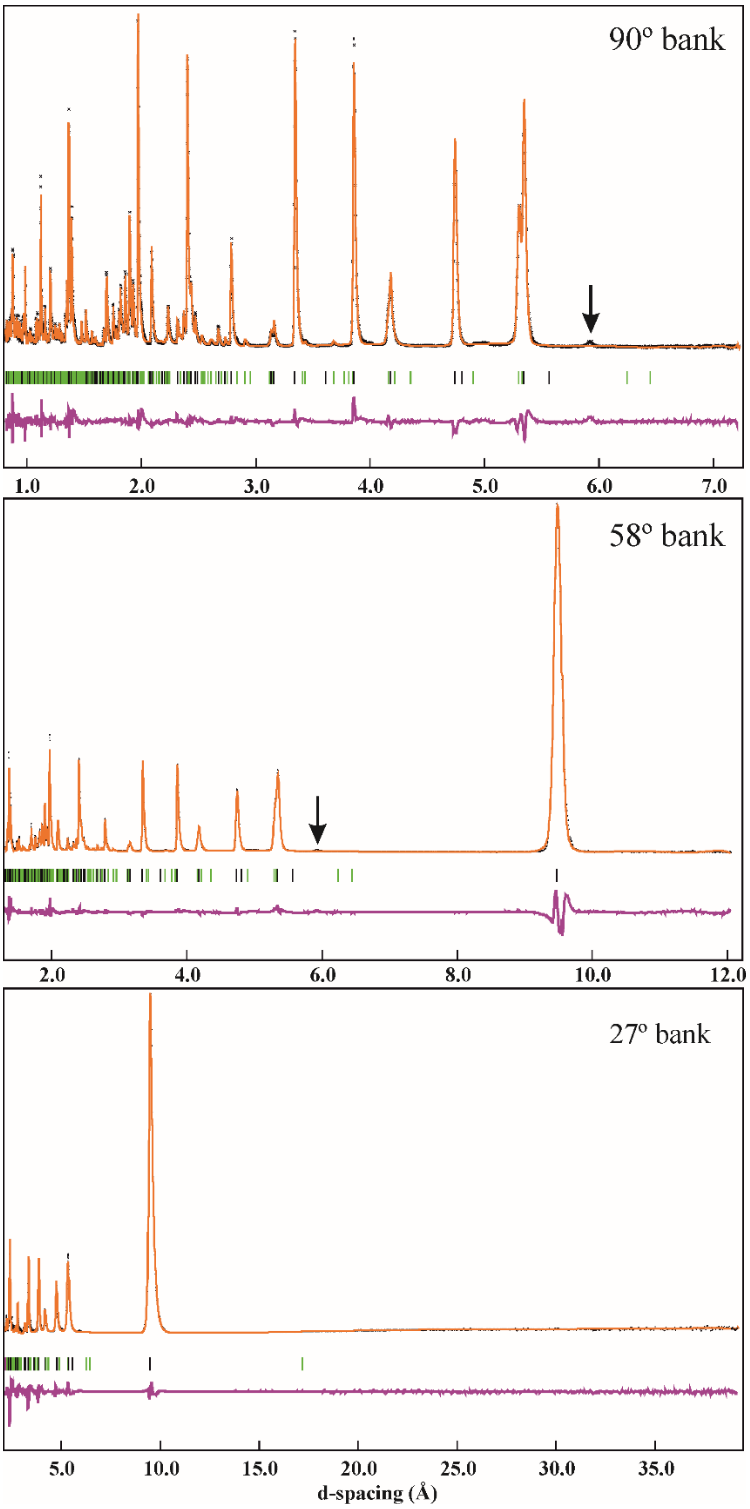

Supplementary Figure 9. Observed, calculated and difference plots from the refinement of an $A2_1a'm'(0\beta 0)000$ model against neutron powder diffraction data collected from $MnSrTa_2O_7$ at 1.5 K. Black and green tick marks indicate positions of main reflections and satellite reflections respectively. Arrows mark peaks from an impurity 'inherited' from the $Li_2SrTa_2O_7$ starting material.

Atom	*x*	*y*	*z*	Fraction	U_{iso} (Å^3)
*Mn(1)	0.4592(18)	0.4967(33)	0.2620(2)	1*	0.0026(18)
	$M_x = 0$	$M_y = 0$	$M_z = 4.064(11)$		
Sr(1)	0.2403(19)	0.2398(9)	0	1	0.0069(6)
Ta(1)	0.7453(14)	0.2501(8)	0.1097(1)	1	0.0070(3)
O(1)	0.7350(21)	0.2261(11)	0	1	0.0195(11)
O(2)	0.7601(12)	0.2721(6)	0.2080(1)	1	0.0072(6)
O(3)	0.9960(12)	0.4986(26)	0.0918(1)	1	0.0070(6)
*O(4')	0.0101(11)	0.0020(17)	0.1184 (1)	1*	0.0028(7)
*O(4'')	0.0101(11)	0.0020(17)	0.0982 (2)	1*	0.0028(7)

$MnSrTa_2O_7$ – magnetic space group $A2_1a'm'(0\beta0)000$, q2 = 0.8639(3)
Δ (Mn) = Δ (O4) = Δ (O4') = 0.5
x_4^0 (Mn) = x_4^0 (Mn) = 0.25, x_4^0 (O4') = 0.75
a = 5.5673(1) Å, *b* = 5.5683(1) Å, *c* = 18.9516(1) Å, volume = 587.506(5) Å^3
Formula weight: 615.85 g mol^{-1}
Radiation source: Neutron Time of flight
Temperature: 1.5 K
GOF = 7.45, wRp = 6.34%, Rp = 5.83%

Supplementary Table 6. Parameters from the structural and magnetic refinement of $MnSrTa_2O_7$ against neutron powder diffraction data collected at 1.5 K.

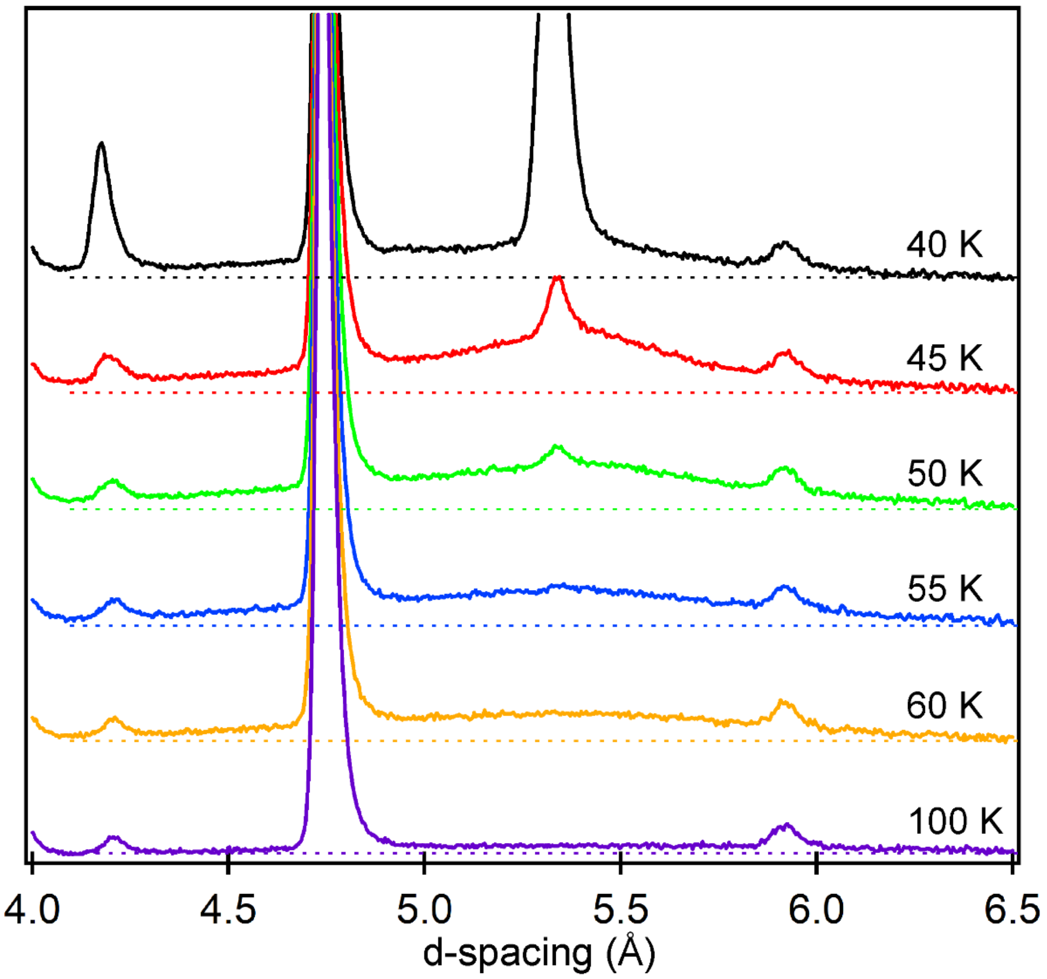

Supplementary Figure 10. NPD data collected from $MnSrTa_2O_7$ highlighting the magnetic diffuse scattering observed above T_N, consistent with the persistence of 2D magnetic correlations above this temperature. Dotted lines are guides to the eye. Peak at ~ 6Å is due to an Li_3TaO_4 impurity is the sample 'inherited' from the $Li_2SrTa_2O_7$ starting material.

To independently confirm the lattice parameter anomaly observed for $MnSrTa_2O_7$ at T ~ 38 K we collected X-ray powder diffraction data as a function of temperature using a Rigaku Smartlab diffractometer fitted with a Ge crystal monochromator (Cu, $K_{\alpha 1}$) and an Oxford Cryosystems Phenix cryostat.

Supplementary Figure 11 shows a fit to a data set collected at 12 K using the commensurate, $A2_1am$ model of $MnSrTa_2O_7$. As shown in Supplementary Figure 4 the satellite peaks are too weak in the X-ray diffraction data to allow a modulated model to be refined. This commensurate model was then refined against a series of data sets collected from $MnSrTa_2O_7$, as a function of temperature, between 20 K and 50 K. As shown in Supplementary Figure 12 the lattice parameter anomalies observed in the NPD data are also observed in the low-temperature X-ray powder diffraction data. It was not possible to extract meaningful changes in the crystal structure of $MnSrTa_2O_7$ from the X-ray diffraction data due to a combination of strong preferred orientation and the weak X-ray scattering powder of the oxide ions, compared to the metal cations (Sr^{2+}, Ta^{5+}).

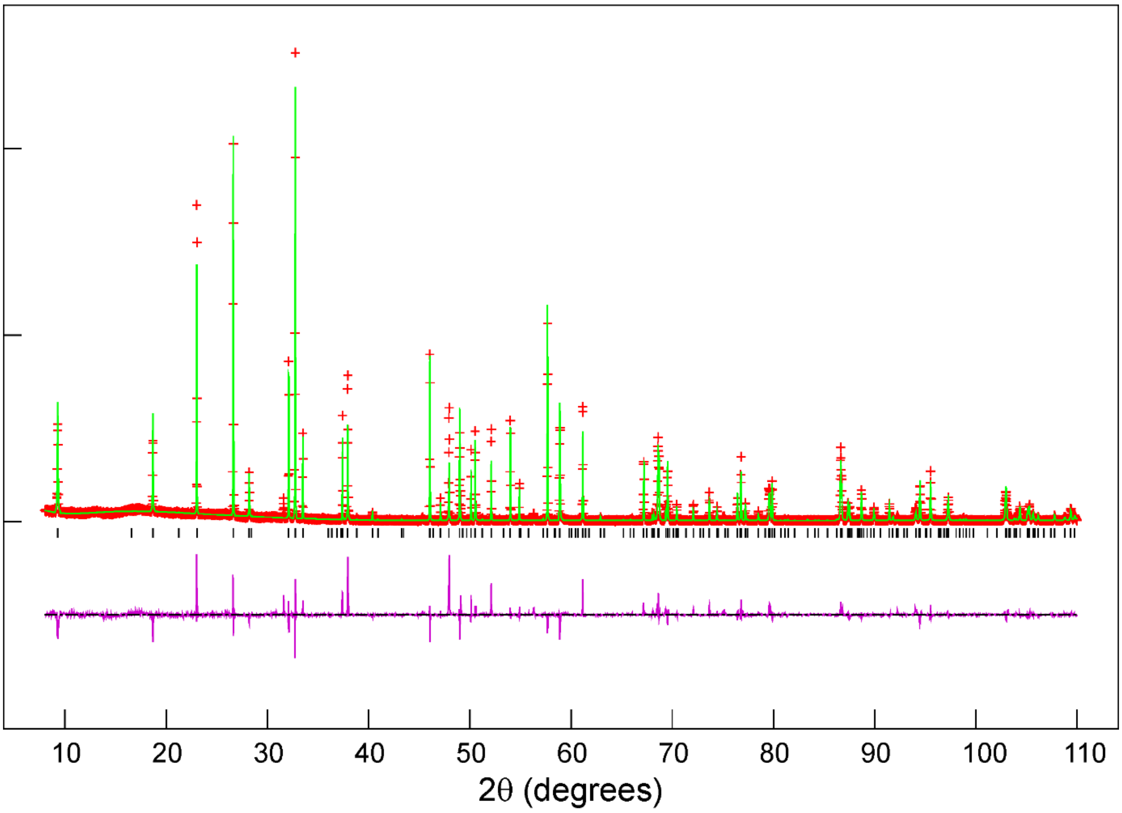

Supplementary Figure 11. Observed calculated and difference plots from a fit to X-ray powder diffraction data collected from $MnSrTa_2O_7$ at 12 K, using a commensurate model (space group $A2_1am$). The poor intensity match between observed and calculated points is attributed to strong preferred orientation exhibited by the material.

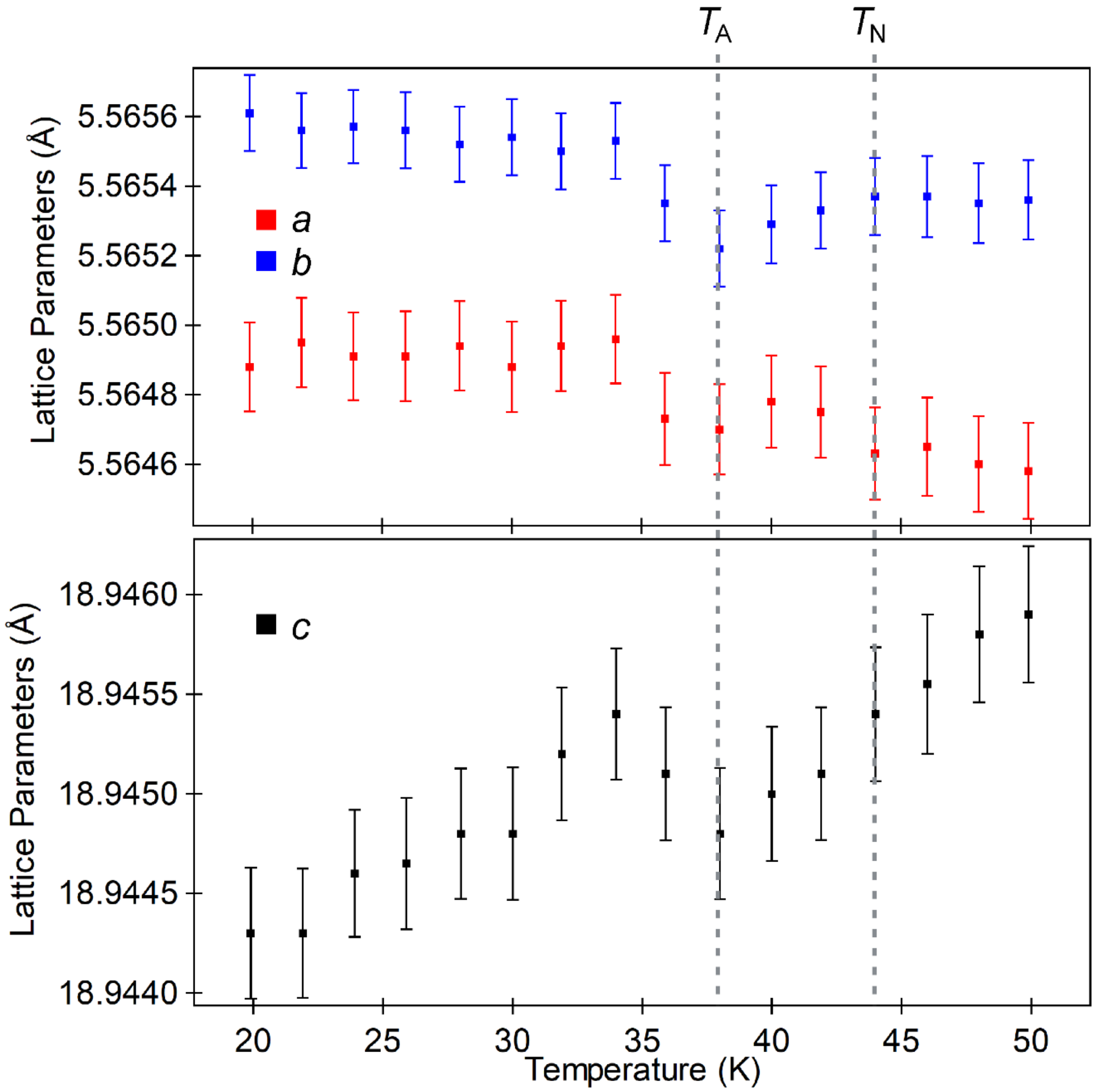

Supplementary Figure 12. A plot of lattice parameters as a function of temperature extracted from fits to X-ray powder diffraction data collected from $MnSrTa_2O_7$.

5. Symmetry analysis and coupling invariants

The symmetry analysis of the magnetic and nuclear structure of $MnSrTa_2O_7$ has been performed with the help of the ISOTROPY suite of software [4,5] using the *I4/mmm* aristotype structure as parent. The analysis shows the presence of four symmetry breaking distortions. These are the two octahedra tilting distortions: X_2^+, described by the two dimensional order parameter (α_1, α_2), that relates to the $a^0a^0c^+/a^0a^0c^+$ tilting and X_3^-, described by the two dimensional order parameter (β_1, β_2), corresponding to the $a^-a^-c^0/a^-a^-c^0$ tilting. Moreover, the incommensurate Mn occupancy distortion, described by the Y_2 mode with order parameter (γ_1, γ_2, γ_3, γ_4), and the antiferromagnetic ordering along the [001] direction described by the mX_3^+ distortion with order parameter (μ_1, μ_2) are observed (all time even irreducible representations will be indicated with an m prefix). These four distortions and the relative primary order parameters are sufficient to reduce the symmetry from the *I*4/*mmm* space group to the observed $A2_1a'm'(0\beta0)000$ magnetic superspace group, as shown in Supplementary Figure 13.

The combination of these four primary order parameters allows the generation of secondary modes thanks to couplings invariant in the Landau free energy. In the following we will derive the couplings that are important in the understanding of the physical properties of $MnSrTa_2O_7$. The free energy invariants will be derived for the general order parameter directions and for the special directions of the $MnSrTa_2O_7$ case.

The combination of the two non-polar tilting distortions allows the generation of a spontaneous electrical polarization through the Hybrid Improper mechanism (HIP). The induced polarization transforms as the Γ_5^- irreducible representation (δ_1, δ_2) and it is due to the trilinear invariant $F_{HIP}=\alpha_1\beta_1\delta_1+\alpha_1\beta_1\delta_2+\alpha_2\beta_2\delta_1-\alpha_2\beta_2\delta_2$, which assuming the high symmetry order parameter directions for X_2^+ (0, α_2) and X_3^- (0, β_2) reduces to $F_{HIP}=\alpha_2\beta_2(\delta_1-\delta_2)$ and constrains the Γ_5^- order parameter along the (δ_1,- δ_1) special direction and further reduces it to the more common trilinear term. The F_{HIP} invariant suggests that the improper ferroelectric polarization, which is directed along one of the [1-10] type directions of the parent *I*4/*mmm* unit cell, can be rotated along the equivalent directions by reversing the sign of either tilting distortion.

It is worth now investigating the symmetry origin of the weak ferromagnetic moment (WFM) to understand the possible coupling with the improper ferroelectricity. The weak ferromagnetic moment transforms as the $m\Gamma_5^+$ (ν_1,ν_2) irreducible representation and gives rise to a net magnetization along one of the [1-10] directions of the parent *I*4/*mmm* cell. The $m\Gamma_5^+$ mode is a secondary mode due to the coupling of the antiferromagnetic mX_3^+ (μ_1, μ_2) mode and the

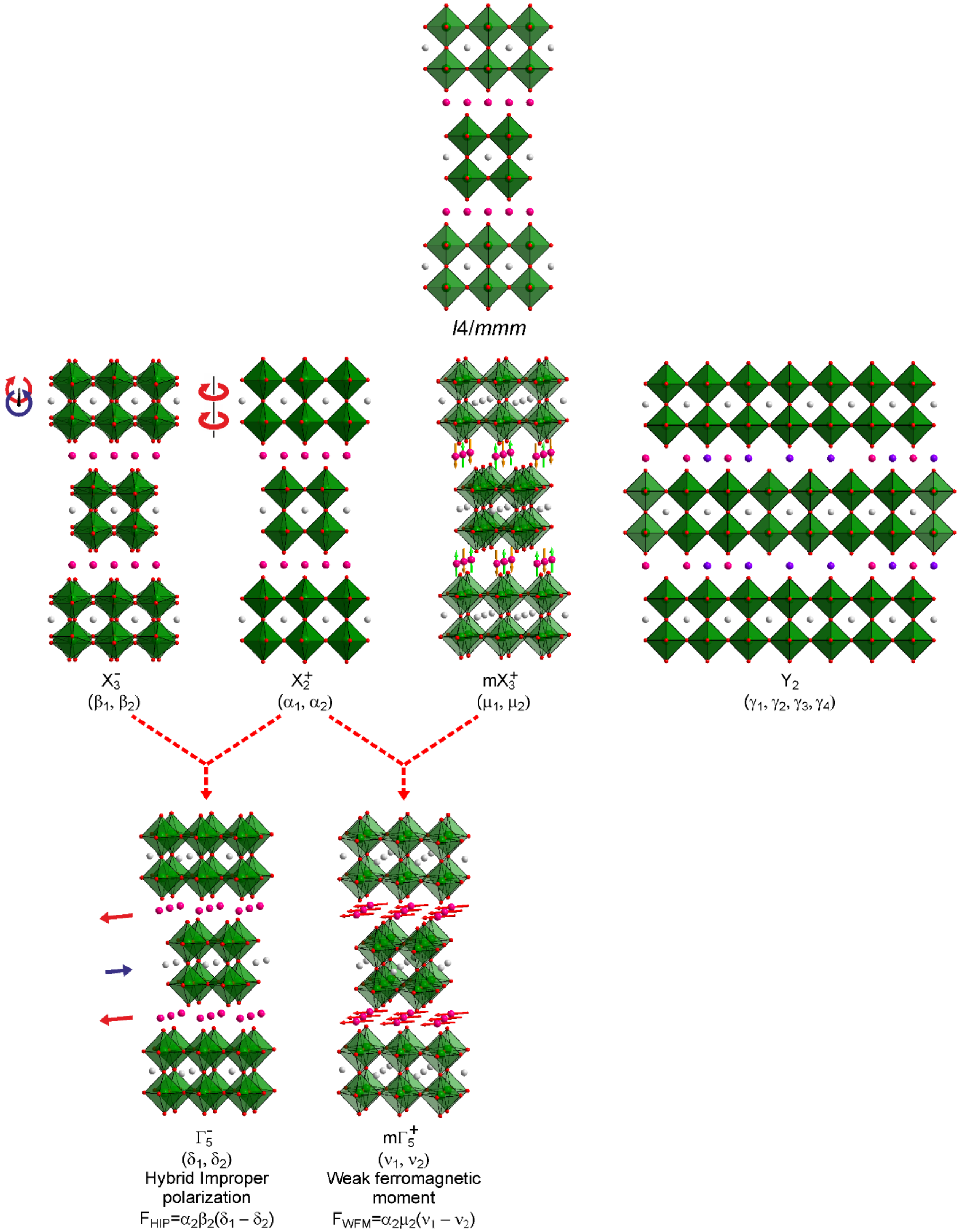

Supplementary Figure 13. The symmetry lowering distortions of $MnSrTa_2O_7$ and their couplings.

X_2^+ (α_1, α_2) tilting. The free energy invariant responsible for the latter coupling is F_{WFM}= $\alpha_1\mu_1\nu_1$+ $\alpha_1\mu_1\nu_2$+ $\alpha_2\mu_2\nu_1$- $\alpha_2\mu_2\nu_2$ which will reduce to $\alpha_2\mu_2(\nu_1- \nu_2)$ if the order parameter directions for $MnSrTa_2O_7$ are considered, and constrain the $m\Gamma_5^+$ order parameter along the (ν_1,-ν_1) direction, as shown in Supplementary Figure 13.

Also in this case the F_{WFM} invariant suggests possible switching paths for the weak ferromagnetic moment which requires either the change of sign of the mX_3^+ antiferromagnetic mode or the X_2^+ octahedral tilting.

These two invariants, F_{HIP} and F_{WFM}, suggest an indirect coupling between the two improper polarizations via the X_2^+ tilting. Indeed, the switching of the improper ferroelectric polarization due to the application of an external electric field will require a change of sign of either the X_3^- or X_2^+ tilting as ruled by F_{HIP}. If we assume that X_2^+ has a lower energy barrier than the X_3^- tilting, then it will be the one to be reversed and as a consequence also either mX_3^+ or the weak ferromagnetic moment need to be reversed as well to keep the system energy unchanged. The latter will likely have a lower energy barrier and hence this provides an electrical control of the weak ferromagnetic moment. The opposite switching path is less likely since an application of an external magnetic field will result in a de-twining of the magnetic domains that correspond to a manipulation of the primary mX_3^+ antiferromagnetic order parameter and not of the X_2^+ tilting.

A direct coupling between the two improper polarizations can also be shown through two fourth degree invariants in the free energy involving the mX_3^+ and X_3^- distortions, which are F_{Coup1}=$\beta_1\mu_1\delta_1\nu_1$+$\beta_1\mu_1\delta_2\nu_2$+$\beta_2\mu_2\delta_1\nu_1$+$\beta_2\mu_2\delta_2\nu_2$ and F_{Coup2}=$\beta_1\mu_1\delta_1\nu_2$+$\beta_1\mu_1\delta_2\nu_1$-$\beta_2\mu_2\delta_1\nu_2$-$\beta_2\mu_2\delta_2\nu_1$. Considering the order parameter directions (0, β_2) for X_3^-, (0, μ_2) for mX_3^+, (δ_1,- δ_1) for Γ_5^- and (ν_1,-ν_1) for $m\Gamma_5^+$ the two coupling invariant reduce to the same F_{Coup}=$\beta_2\mu_2\delta_1\nu_1$. The latter coupling term clearly indicates that the common change of the two improper polarizations leaves the energy of the system invariant rigorously showing the coupling between the two improper ferroic orders in $MnSrTa_2O_7$.

References

1 Pagnier, T. *et al.* Phase transition in the Ruddlesden-Popper layered perovskite $Li_2SrTa_2O_7$. *J. Solid State Chem.* **182**, 317-326 (2009).

2 van Smaalen, S. *Incommensurate Crystallography*. (Oxford University Press, 2007).

3 Petricek, V., Vanderlee, A. & Evain, M. On the use of Crenel functions for occupationally modulated structures. *Acta Cryst. Sect. A* **51**, 529-535 (1995).

4 ISOTROPY Software Suite (iso.byu.edu, 2007).

5 Campbell, B. J., Stokes, H. T., Tanner, D. E. & Hatch, D. M. ISODISPLACE: a web-based tool for exploring structural distortions. *J. Appl. Crystallogr.* **39**, 607-614 (2006).
